# Phloroglucinol Enhances Anagen Signaling and Alleviates H_2_O_2_-Induced Oxidative Stress in Human Dermal Papilla Cells

**DOI:** 10.4014/jmb.2311.11047

**Published:** 2024-03-08

**Authors:** Seokmuk Park, Ye Jin Lim, Hee Su Kim, Hee-Jae Shin, Ji-Seon Kim, Jae Nam Lee, Jae Ho Lee, Seunghee Bae

**Affiliations:** 1 Department of Cosmetics Engineering, Konkuk University, Seoul 05029, Republic of Korea; 2 Department of Cosmetology, Graduate School of Engineering, Konkuk University, Seoul 05029, Republic of Korea

**Keywords:** Phloroglucinol, hair growth, hair loss, oxidative stress, protein kinase B (AKT), Human dermal papilla cells (HDPCs)

## Abstract

Phloroglucinol (PG) is one of the abundant isomeric benzenetriols in brown algae. Due to its polyphenolic structure, PG exhibits various biological activities. However, the impact of PG on anagen signaling and oxidative stress in human dermal papilla cells (HDPCs) is unknown. In this study, we investigated the therapeutic potential of PG for improving hair loss. A non-cytotoxic concentration of PG increased anagen-inductive genes and transcriptional activities of β-Catenin. Since several anagen-inductive genes are regulated by β-Catenin, further experiments were performed to elucidate the molecular mechanism by which PG upregulates anagen signaling. Various biochemical analyses revealed that PG upregulated β-Catenin signaling without affecting the expression of Wnt. In particular, PG elevated the phosphorylation of protein kinase B (AKT), leading to an increase in the inhibitory phosphorylation of glycogen synthase kinase 3 beta (GSK3β) at serine 9. Treatment with the selective phosphoinositide 3-kinase/AKT inhibitor, LY294002, restored the increased AKT/GSK3β/β-Catenin signaling and anagen-inductive proteins induced by PG. Moreover, conditioned medium from PG-treated HDPCs promoted the proliferation and migration of human epidermal keratinocytes via the AKT signaling pathway. Subsequently, we assessed the antioxidant activities of PG. PG ameliorated the elevated oxidative stress markers and improved the decreased anagen signaling in hydrogen peroxide (H_2_O_2_)-induced HDPCs. The senescence-associated β-galactosidase staining assay also demonstrated that the antioxidant abilities of PG effectively mitigated H_2_O_2_-induced senescence. Overall, these results indicate that PG potentially enhances anagen signaling and improves oxidative stress-induced cellular damage in HDPCs. Therefore, PG can be employed as a novel therapeutic component to ameliorate hair loss symptoms.

## Introduction

Hair is a distinctive feature of mammals and provides several key advantages for maintaining skin homeostasis, including thermoregulation, sebum production, and protection against ultraviolet radiation [1]. Hair follicles undergo a specific growth cycle comprising three distinct phases (anagen, catagen, and telogen) to maintain tissue homeostasis [2]. The critical components of the hair follicle include hair follicle stem cells (hfSCs) and dermal papilla cells (DPCs), which primarily regulate the hair cycle through intricate signaling interactions [3-5]. DPCs are responsive to multiple signaling pathways, including wingless (Wnt) and sonic hedgehog (Shh), and secrete proteins, such as fibroblast growth factor (FGF)7, FGF10, and Noggin (NOG) [6, 7]. These secreted proteins have pivotal roles in promoting the differentiation and proliferation of hfSCs, ultimately triggering the telogen-anagen transition in hair follicles [8]. Recent studies have been focused on developing therapeutic candidates to prevent hair loss by employing strategies that activate Wnt or Shh signaling pathways within DPCs [2, 9-11].

Alopecia, commonly known as hair loss, involves the shedding and miniaturization of hair follicles [12]. While hair loss may not directly threaten survival, it significantly impacts an individual's quality of life by causing psychological distress [13-15]. Prominent factors contributing to alopecia involve genetic elements, androgens, stress, and inflammation; however, the precise molecular mechanisms remain unclear [16-19]. Previous studies have indicated that DPCs derived from patients with androgenetic alopecia (AGA) exhibit slowed growth and premature senescence [20]. Furthermore, a growing body of evidence suggests that oxidative stress and premature senescence in DPCs can trigger the catagen phase of hair follicles, implicating novel etiological factors in hair loss [21]. Efforts are increasing to alleviate hair loss by suppressing oxidative stress and cellular senescence [22, 23]. Dexpanthenol, a well-established ingredient in hair care products, is reportedly effective in preventing hair loss by inhibiting cellular senescence and apoptosis in human dermal papilla cells (HDPCs) [24].

The only drugs approved by the United States Food and Drug Administration for treating hair loss are minoxidil, finasteride, and baricitinib [25]. However, due to the occurrence of side effects in several patients, there is a compelling need for the development of natural therapeutic agents to alleviate hair loss [26, 27]. Phloroglucinol (PG) and its derivatives are secondary metabolites naturally isolated from various plants and algae species [28-30]. PG possesses several pharmacological potentials, including antibacterial, anti-inflammatory, and anti-allergic properties [31-34]. Furthermore, PG can protect human keratinocytes from DNA damage and cellular apoptosis induced by oxidative stress [35]. Several studies have also suggested that PG can protect the skin from damage caused by ultraviolet B radiation, suggesting the possibility of PG as a therapeutic agent derived from natural sources [36-38]. However, the effects of PG on HDPCs and its potential impact on hair loss have not been previously investigated. Thus, our study aims to elucidate, for the first time, how PG enhances anagen signaling and alleviates oxidative stress in HDPCs.

## Materials and Methods

### Chemicals and Cell Culture

Human hair follicle dermal papilla cells (HDPCs; #C-12071; PromoCell, Germany) were purchased and were used in this study between passages 4 and 7. The cells were maintained at 37°C and 5% CO_2_ using a follicle growth medium kit (#C-26051; PromoCell). Additionally, 293T cells (#CRL-3216) and human epidermal keratinocyte cell line (HaCaT; #300493) were procured from American Type Culture Collection (USA) and German Cancer Research Center (Germany), respectively. They were maintained in Dulbecco’s modified Eagle’s medium (#LB001-05; Welgene, Republic of Korea) supplemented with 10% (v/v) fetal bovine serum (#35-015-CV; Gibco; Thermo Fisher Scientific, USA) and 1% penicillin/streptomycin (#15140-122; Gibco; Thermo Fisher Scientific). Phloroglucinol (PG) was purchased from Tokyo Chemical Industry Co. (#P0249; Japan). LY294002 (#440202), MG132 (#474701), dihydrotestosterone (DHT; #A8380), and cycloheximide (CHX; #01810) were obtained from Sigma-Aldrich (Merck KGaA, USA).

### Cell Viability Assay

The cytotoxicity of PG in HDPCs and HaCaT cells was assessed through the water-soluble tetrazolium salt (WST-1) assay, utilizing the EZ-Cytox Cell Viability Assay Kit (#EZ3000; DoGenBio, Republic of Korea). HDPCs (2 × 10^3^) and HaCaT cells (5 × 10^3^) were seeded in a 96-well plate and incubated at 37°C for 24 h. Subsequently, the cells were treated with each drug for 48 h. The supernatant was removed, 100 μl of EZ-cytox solution was added to each well, and the mixture was incubated at 37°C for 30 min. Cell viabilities were determined by measuring the absorbance at 450 nm using a Synergy HTX Multi-Mode Microplate Reader (Bioteck, USA).

### Intracellular ROS Measurement

The efficacy of PG in inhibiting intracellular ROS level was assessed using 2’,7’-dichlorofluorescein diacetate (H_2_DCFDA; #D6883; Sigma-Aldrich; Merck KGaA), following the method described by Ng in 2021 [39, 40]. HDPCs (5 × 10^3^) were seeded in a 96-well fluorescence microtiter plate and incubated at 37°C for 24 h. Subsequently, the cells were treated with various concentrations of PG (0-10 μM) for 60 min. The PG-treated cells were washed with Dulbecco’s phosphate-buffered saline (DPBS; #LB001-02; Welgene) and the plate was loaded with 10 μM H2DCFDA at 37°C for 30 min. To induce intracellular ROS generation, the cells were washed with DPBS and then treated with 1 mM hydrogen peroxide (H_2_O_2_; #4104-4405; DaejungChemicals, Republic of Korea) for 10 min at 37°C. Afterwards, intracellular ROS levels were quantified by measuring 2’,7’-dichlorofluorescein using a Synergy HTX Multi-Mode Microplate Reader (Bioteck) with excitation at 485 nm and emission at 520 nm.

### TOPFlash Luciferase Reporter Assay

The TOPFlash luciferase reporter assay was conducted following the method described by S. Park *et al*. [40]. 293T cells (5 × 10^5^) were seeded in 6-well plates and incubated at 37°C for 24 h. The cells were transfected with pSV-β-galactosidase plasmid (#E1081; Promega, USA) and TCF/LEF response element-driven luciferase reporter plasmid (#12456; Addgene, USA) using Lipofectamine transfection reagent (#18324012; Invitrogen; Thermo Fisher Scientific). The transfected cells were treated with PG (5 or 10 μM) or 5 mM lithium chloride (#L9650; Sigma-Aldrich; Merck KGaA) at 37°C for 24 h. Then, the cells were lysed with passive lysis 5× buffer (#E1941; Promega) for 30 min 4°C. Cell lysate was harvested following centrifugation at 15,000 ×g for 30 min at 4°C. D-luciferin (#88294; Invitrogen; Thermo Fisher Scientific) and cell lysate were reacted at 37°C for 30 min, and then luciferase activity was measured using a microplate reader. β-Galactosidase activity was assessed utilizing the Beta-Glo Assay System (#E4720; Promega), and relative luciferase activity was normalized to β-galactosidase activity.

### Conditioned Medium Preparation

HDPCs (1 × 10^5^) were seeded in a 6-well plate and incubated at 37°C for 24 h. Then, the cells were treated with PG (0-10 μM) in the presence or absence of LY294002 (0-20 μM) for 24 h. After treatment, the attached HDPCs were gently washed with DPBS and the medium was replaced with serum-free DMEM for 24 h. Subsequently, conditioned medium (CM) was collected, centrifuged at 2,000 rpm for 10 min, and filtered through a 0.2 μm syringe filter (#S6534; Sartorius, Germany) to remove cell debris. The prepared CM was utilized for the treatment of HaCaT cells, and CM-treated HaCaT cells were used for proliferation and migration analyses.

### Clonogenic Assay

To evaluate the proliferative effects of CM from PG-treated HDPCs on HaCaT cells, a clonogenic assay based on crystal violet staining was performed. HaCaT cells (3 × 10^4^) were seeded in 24-well plates and incubated for 24 h. Subsequently, various types of CM were added, and the cells were further incubated for 48 h. After the 48 h incubation, the cells were washed twice with 500 μl of DPBS. The residual DPBS was then aspirated to ensure complete drying, and each well was stained with a 0.5% crystal violet (#6408; Biopure, Republic of Korea) in 20%methanol for 2 h. Following staining, the plate was rinsed ten times with distilled water and allowed to air dry. The crystal violet solution within the cells was completely solubilized using a 100% methanol solution. Proliferation rates were determined by measuring the absorbance at 570 nm using a Synergy HTX Multi-Mode Microplate Reader (Bioteck).

### In Vitro Wound Healing Assay

For migration assessment, a wound healing assay was conducted by generating scratches using a conventional pipette tip. HaCaT cells (3 × 10^5^) were plated in 12-well plates and incubated at 37°C until reaching 100%confluence. The confluent cell layer was then scratched with a sterile plastic 20 μl pipette tip and gently washed twice with DPBS. Subsequently, the cells were cultured with the indicated types of CM for 36 h. Cell migration into the wound area was photographed at 12 h intervals. Images were captured using a low magnification (4×) objective of a bright-field microscope, and the gap area was quantified using the ImageJ software version 1.53t (National Institutes of Health, USA).

### SA-β-Gal-Based Cellular Senescence Analysis

The analysis of cellular senescence was conducted by staining of β-galactosidase, a well-known aging marker, following the method described by Florence *et al*. (2009) [41]. HDPCs (1 × 10^4^) were seeded in 12-well plates and incubated at 37°C for 24 h. Subsequently, the cells were pretreated with PG (2, 5, or 10 μM) for 30 min and then washed with DPBS. Following this, the cells were treated with 0.1 mM H_2_O_2_ for 72 h at 37°C. After removing the supernatant, the cells were washed twice with DPBS and fixed using 4% formaldehyde (#F8775; Sigma-Aldrich; Merck KGaA) for 15 min at room temperature. The cells were then stained using the senescence β-galactosidase staining kit (#9860S; Cell Signaling Technology, USA) for 24 h at 37°C. Senescent cells were counted using a bright-field microscope, and the percentages were analyzed.

### Quantitative Real-Time PCR

For quantitative real-time PCR, HDPCs (5 × 10^5^) were seeded in 100 mm dishes and incubated at 37°C for 24 h. Then, cells were treated with PG (0-10 μM) in the presence or absence of 0.1 mM H_2_O_2_ for 12 h. Total RNA extraction process used RiboEx reagent (#301-001; Geneall Biotechnology, Republic of Korea), and all procedures were carried out following the manufacturer's instructions. The complementary DNA was synthesized using 1 μg of extracted total RNA, 2.5 mM dNTPs, oligo dT primers, 0.1 M DTT, 5X Firse-Strand Buffer, and M-MLV reverse transcriptase (#28025013; Invitrogen; Thermo fisher scientific). Quantitative real-time PCR was performed using SYBR Green PCR Master Mix (#4309155; Invitrogen; Thermo Fisher Scientific), primers, and cDNA. The expression level of mRNA was normalized by glyceraldehyde-3-phosphate dehydrogenase (*GAPDH*) and analyzed with the StepOnePlus Real-Time PCR system (Thermo Fisher Scientific). In this experiment, the sequences of primers are provided in Table 1.

### Western Blot Analysis

HDPCs (5 × 10^5^) were seeded in 100 mm dishes and incubated at 37°C for 24 h. Then, cells were treated with PG (0-10 μM) in the presence or absence of 20 μM LY294002, 40 μM CHX, 5 μM CHIR99021, 0.1 mM H_2_O_2_, or 2 μM MG132 at 37°C. The drug-treated cells were lysed using cell lysis buffer (#4719964001; Roche; Merch KGaA) containing phosphatase inhibitor cocktail (#4906845001; Roche; Merch KGaA). The protein concentration was quantified using Pierce BCA Protein Assay Kit (#23225; Thermo Fisher Scientific). Twenty micrograms of protein lysates were separated by sodium dodecyl sulfate-polyacrylamide gel electrophoresis and were transferred to nitrocellulose membranes (#88018; Thermo Fisher Scientific). The membranes were incubated with the corresponding primary antibodies overnight at 4°C, and then horseradish peroxidase-conjugated anti-mouse IgG (#7076S; Cell Signaling Technology; CST; USA) or anti-rabbit IgG (#7074S; CST) secondary antibodies were incubated for 2 h at room temperature. The blots were detected using Clarity Western ECL substrates (#1705061; Bio-Rad Laboratories, USA) and the intensities of the protein bands were analyzed with ImageJ software version 1.53t (National Institutes of Health). The following primary antibodies and dilution are listed in Table 2.

### Statistical Analysis

In this study, all statistical analyses were conducted on at least three independent experiments. One-way analysis of variance (ANOVA) was employed to evaluate the statistical differences among the various groups using the GraphPad Prism software (version 8.0.1, USA). In cases where statistical significance was detected, Tukey's test was utilized to compare the means of multiple groups within each treatment category. The data are presented as mean values ± standard deviation (SD), and significance between groups was determined using a *p*-value threshold of less than 0.05.

## Results

### PG Increases Hair-Inductive Genes with No Cytotoxicity in HDPCs

**Fig. 1:**
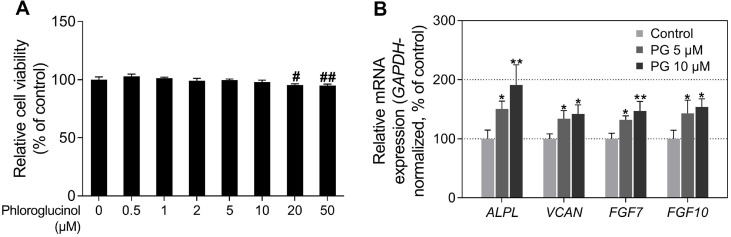
Effects of phloroglucinol (PG) on cell viability and expression of anagen-inductive genes in human dermal papilla cells (HDPCs). (**A**) HDPCs are treated with PG (0-50 μM) for 48 h, and cell viabilities are assessed using a water-soluble tetrazolium salt-1 (WST-1) assay. (**B**) The expression levels of anagen-inductive genes (*ALPL*, *VCAN*, *FGF7*, and *FGF10*) are analyzed by quantitative real-time polymerase chain reaction (qRT-PCR) and normalized against GAPDH. The cells are treated with either 5 or 10 μM of PG for 12 h. The results are presented as the mean ± SD of three independent experiments and are analyzed using a one-way analysis of variance followed by Tukey’s test. ^#,*^p < 0.05; ^##,**^p < 0.01 compared with the vehicle-treated group.

PG and its derivatives are secondary metabolites found in various microorganisms, including marine brown and red algae [42]. The compounds are primarily characterized by 1,3,5-trihydroxybenzene as their basic moiety and possess numerous bioactivities [43]. In particular, 7-phloroeckol, one of the PG derivatives reportedly promotes hair follicle elongation and enhances the expression of hair growth-related genes [44]. However, whether PG regulates the expression of hair-inductive genes in HDPCs and its molecular mechanism have not been reported to date. Before performing various biochemical analyses involving HDPCs, the potential cytotoxicity of PG was evaluated. No cytotoxic effect was observed in HDPCs treated with PG doses below 10 μM PG (Fig. 1A). Consequently, doses of up to 10 μM PG were selected for subsequent experiments.

In previous studies, it was reported that PG derivatives enhance the expression of hair growth-related genes [45]. Therefore, we investigated whether PG regulates the expression of hair-inductive genes. Alkaline phosphatase (*ALPL*) and versican (*VCAN*) are signature genes that exhibit specifically elevated expression within DPCs during the anagen phase of hair follicles [46, 47]. FGF7 and FGF10 secreted from DPCs are core secretory proteins that induce the anagen phase of telogenic hair follicles [48, 49]. As shown in Fig. 1B, PG upregulated the mRNA expressions of *ALPL*, *VCAN*, *FGF7*, and *FGF10* in a dose-dependent manner. In particular, the cells treated with 10 μM PG exhibited a 190.85, 141.92, 146.97, and 153.83% increase in *ALPL*, *VCAN*, *FGF7*, and *FGF10*, respectively (Fig. 1B). These results suggest that similar to other PG derivatives, PG also upregulates the hair-inductive genes, indicating its potential as a novel therapeutic candidate for promoting hair growth.

### PG Activates β-Catenin Signaling Pathway without Regulating Canonical WNTs Expression in HDPCs

**Fig. 2:**
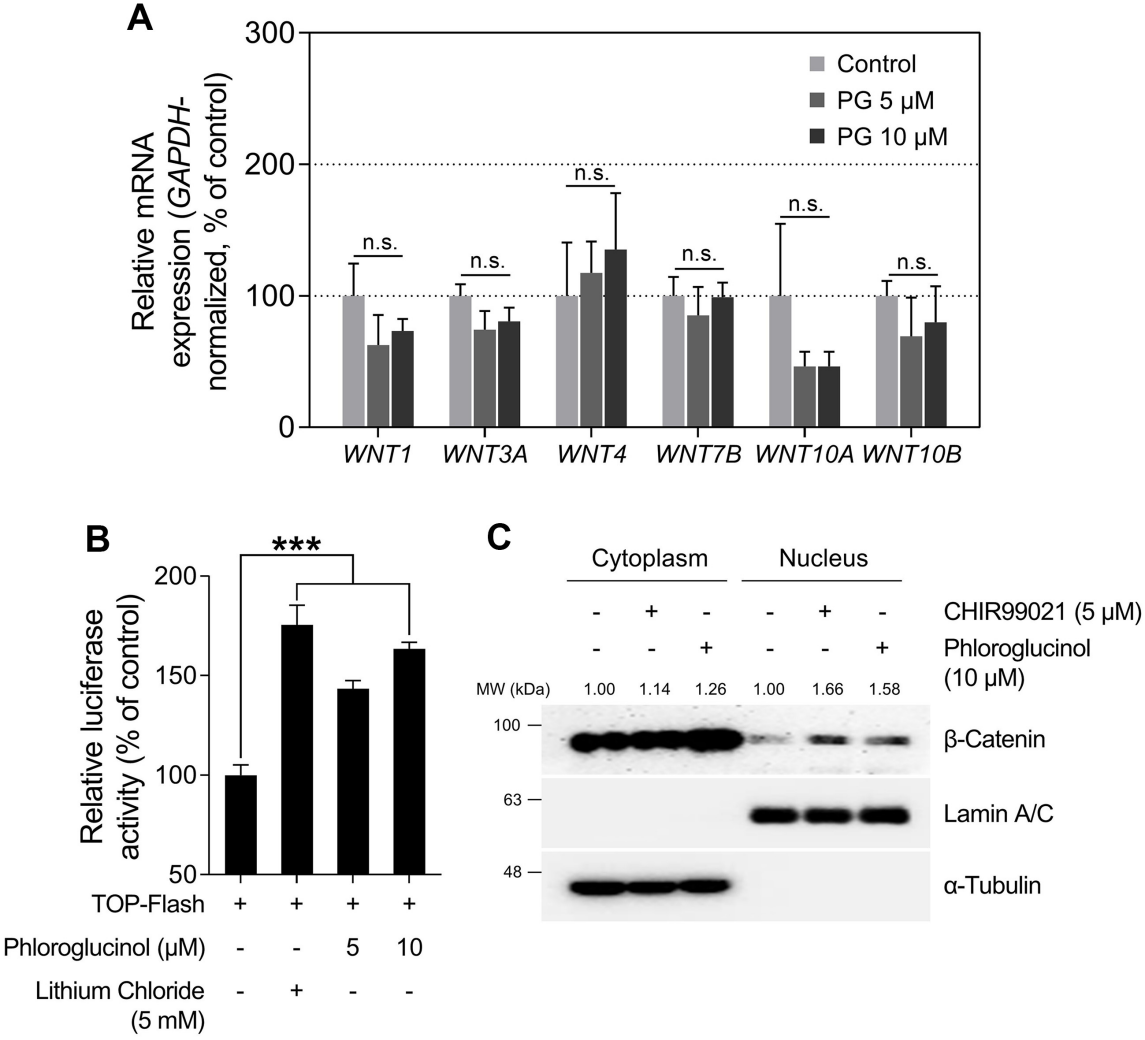
Effects of phloroglucinol (PG) on WNT/β-Catenin signaling pathway in HDPCs. (**A**) The expression levels of *WNT* genes (*WNT1*, *WNT3A*, *WNT4*, *WNT7B*, *WNT10A*, and *WNT10B*) are determined by qRT-PCR and normalized against *GAPDH*. HDPCs are treated with either 5 or 10 μM of PG for 12 h. (**B**) 293T cells are treated with PG (5 or 10 μM) or lithium chloride (5 mM) for 24 h. TOP-Flash luciferase reporter luminescence assay has been performed and normalized against β-galactosidase activity. (**C**) The nuclear translocation of β-Catenin is analyzed by western blotting. α- Tubulin and Lamin A/C served as loading controls for the cytoplasmic and nuclear fractions, respectively. The results are presented as the mean ± SD and significant differences are analyzed using a one-way analysis of variance followed by Tukey’s test. ***p < 0.001 compared with the vehicle-treated group.

The regulation of hair inductive properties has been reported to be closely associated with the Wingless-related integration site (WNT)/β-Catenin signaling pathway in DPCs [50]. Several anagen-inductive genes, including *ALPL*, are primarily regulated by β-Catenin signaling [51, 52]. Therefore, we investigated whether PG activates the β-Catenin signaling pathway and sought to elucidate its regulatory mechanism in HDPCs. In previous studies, the canonical WNT pathway has been predominantly discussed as a major signaling pathway that enhances the transcriptional activity of β-Catenin [53]. In the presence of canonical WNT ligands, they bind to the Frizzled receptor and lipoprotein receptor-related protein, thereby triggering a β-Catenin signaling cascade [53]. Ultimately, β-Catenin is released from the destruction complex, leading to increased cytoplasmic stabilization [54]. Canonical WNT ligands, such as WNT1, WNT3A, WNT4, WNT7B, WNT10A, and WNT10B, have been reported to promote hair growth through the upregulation of β-Catenin signaling [55-62]. In contrast to β-Catenin, whose protein stability is primarily regulated through the ubiquitin proteasome system (UPS), WNT ligands are notably controlled at the transcriptional and post-transcriptional level [63, 64]. Once translated into proteins, WNT ligands require posttranslational modification to be secreted outside the cell. This process crucially involves palmitoylation by porcupine and intracellular trafficking facilitated by the carrier protein Wntless [65-67]. Therefore, we investigated whether PG regulates the expression of *WNT*, influencing the protein activity of β-Catenin. We observed that PG did not affect the mRNA expression of *WNT* ligands, which are key regulators of the β-Catenin signaling pathway, but it upregulated the transcriptional activity of β-Catenin (Fig. 2A-2C) [68]. As shown in Fig. 2B, PG stimulated T cell factor/lymphoid enhancer-binding factor (TCF/LEF)-driven luciferase activity, resulted in 143.47 and 163.45% increases at PG doses of 5 and 10 μM, respectively. Furthermore, nuclear fractionation revealed an increase in β-Catenin translocation into the nucleus upon treatment with 10 μM PG compared to that of untreated control (Fig. 2C). Collectively, these results suggest that PG does not alter the expression of canonical Wnt ligands but increases the transcriptional activity of β-Catenin.

### PG Activates β-Catenin Signaling Pathway by Inhibiting Proteasome-Mediated β-Catenin Degradation.

**Fig. 3:**
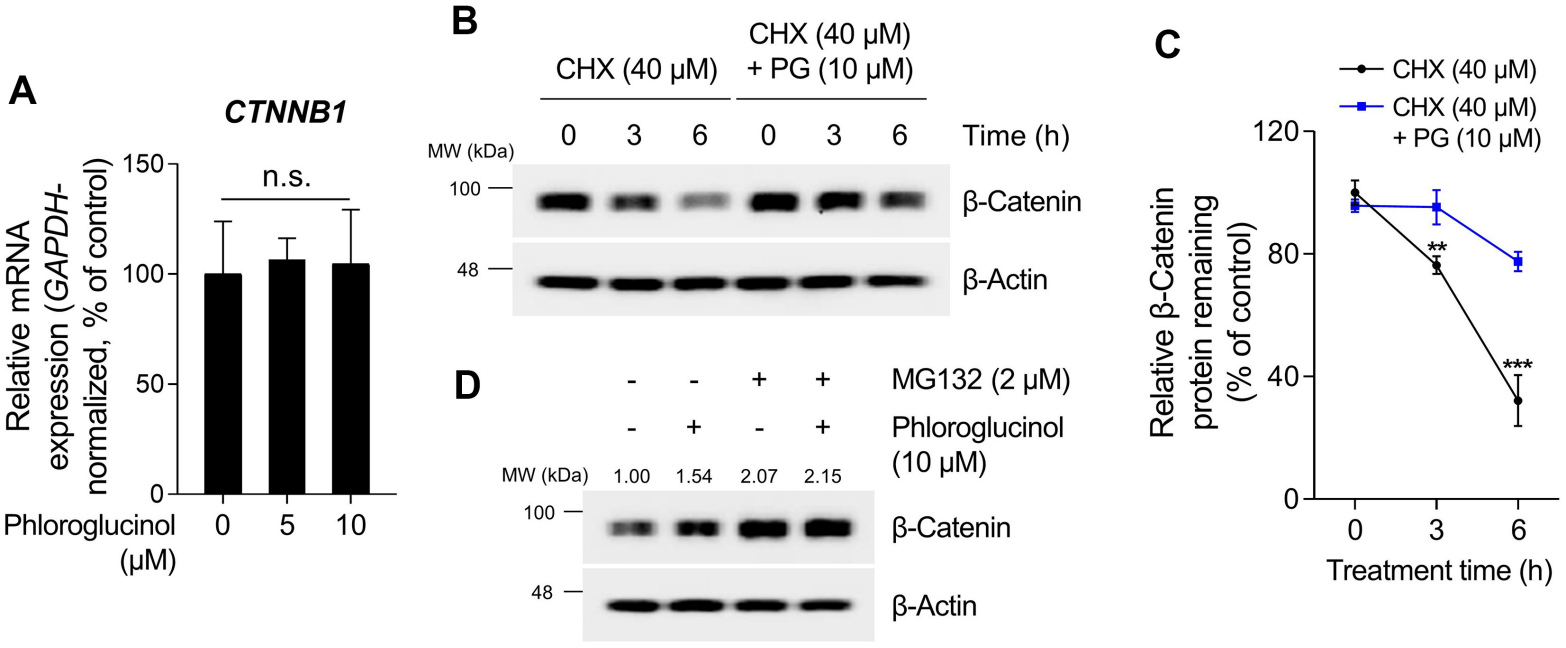
Effects of phloroglucinol (PG) on the regulation of β-Catenin proteostasis in HDPCs. (**A**) The mRNA expression levels of *CTNNB1* are determined by qRT-PCR and normalized against *GAPDH*. HDPCs are treated with either 5 or 10 μM of PG for 12 h. (**B-C**) The protein stability of β-Catenin is assessed at various time points following treatment with PG (10 μM) with or without cycloheximide (CHX) (40 μM) in HDPCs. (**D**) HDPCs are treated with PG (10 μM) with or without MG132 (2 μM) for 12 h, and protein levels of the β-Catenin are analyzed by western blotting, with β-Actin serving as a loading control. Quantification of protein levels is carried out using ImageJ software version 1.53t. The results are presented as the mean ± SD and significant differences are analyzed using a one-way analysis of variance followed by Tukey’s test. **p < 0.01; ***p < 0.001 compared with the vehicle-treated group.

The protein stability of the transcription coactivator β-Catenin is primarily regulated by ubiquitination mediated by the ubiquitin E3 ligase β-TrCP, resulting in subsequent proteasomal degradation [69]. Specifically, within the destruction complex, CK1α phosphorylates Ser45, while GSK3 phosphorylates Ser33, Ser37, and Thr41 of β-Catenin. These phosphorylation sites subsequently induce β-TrCP-mediated proteolysis of β-Catenin [70]. Hence, we examined whether PG enhances β-Catenin stability by inhibiting the proteasome pathway. As depicted in Fig. 3A, PG did not induce any changes in the mRNA level of *β-Catenin* (Fig. 3A). Subsequent experiments were conducted to confirm whether PG, which does not alter *β-Catenin* mRNA levels, increases the protein half-life of β-Catenin. Cells treated with the protein synthesis inhibitor cycloheximide (CHX) exhibited a significant decrease in protein stability of β-Catenin (Fig. 3B-3C). However, in cells co-treated with PG (10 μM) and CHX (40 μM), the protein half-life of β-Catenin increased compared to those treated with CHX alone (Fig. 3B-3C). These results suggest that PG regulates β-Catenin signaling by increasing protein stability rather than enhancing gene expression.

Protein homeostasis, also referred to as proteostasis, is typically regulated through the UPS or lysosomal degradation pathways [71]. Previous studies have indicated that ubiquitin-mediated proteasomal degradation serves as the primary regulatory pathway for maintaining the proteostasis of β-Catenin [72]. Therefore, we verified whether the increase in protein half-life induced by PG is mediated by the proteasomal inhibition. As shown in Fig. 3D, the protein stability of β-Catenin was significantly enhanced in cells treated with MG132 (2 μM) or PG (10 μM) alone. In contrast, the stability of β-Catenin did not exhibit further enhancement when PG was co-administered with MG132. Therefore, these results suggest that PG activates the β-Catenin signaling pathway by inhibiting proteasome-mediated β-Catenin degradation.

### PG Enhances Anagen Signaling by Activating AKT/GSK3β/β-Catenin Signaling Pathway in HDPCs

**Fig. 4:**
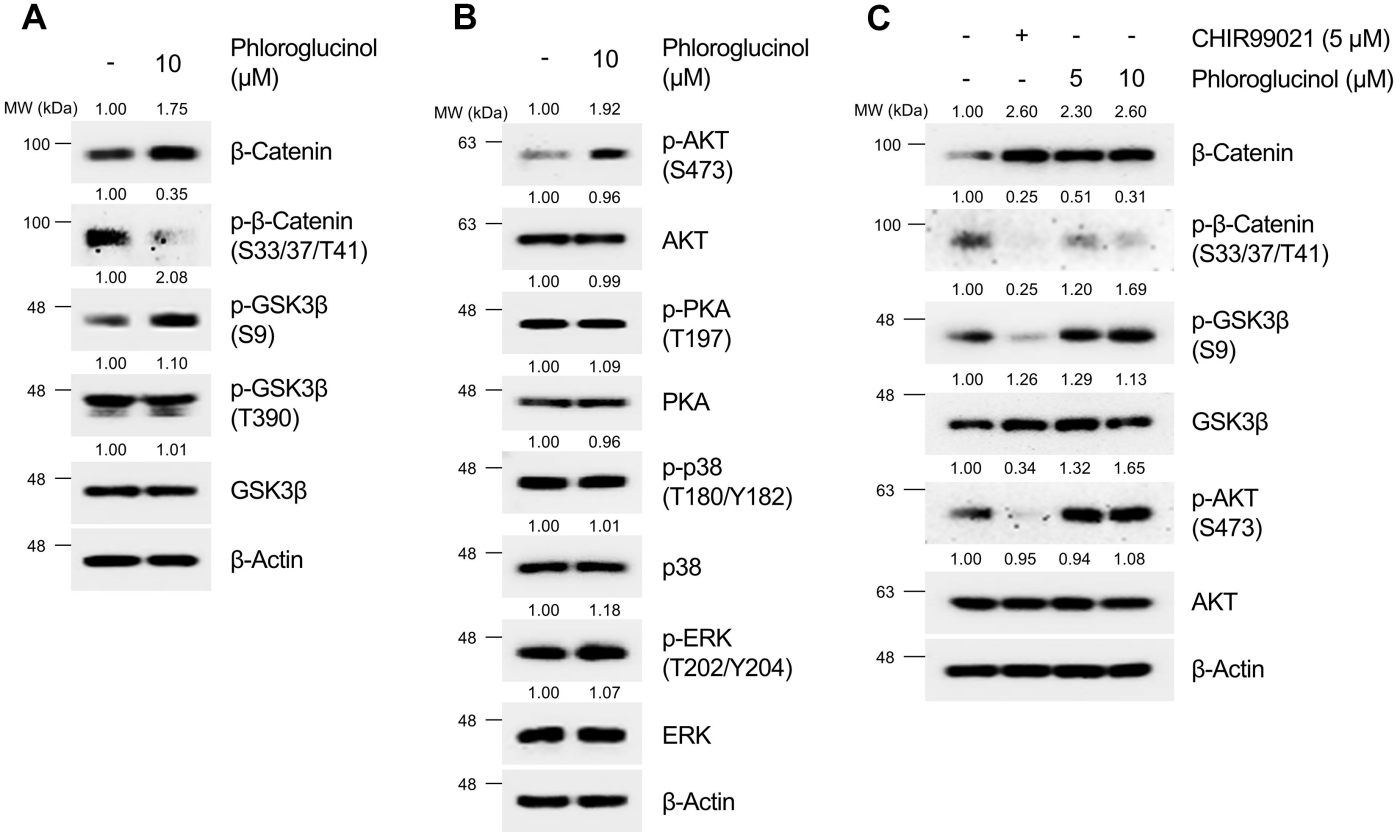
Effects of phloroglucinol (PG) on the inhibitory phosphorylation of glycogen synthase kinase 3 beta (GSK3β) and its upstream signaling pathways in HDPCs. (**A-B**) HDPCs were treated with 10 μM PG for 12 h. Protein levels of β-Catenin and the inhibitory phosphorylation of GSK3β, along with its upstream kinases, are analyzed by western blotting, with β-Actin serving as a loading control. (**C**) HDPCs are treated with either PG (5 or 10 μM) or CHIR99021 (5 μM) for 24 h, and protein levels of the protein kinase B (AKT)/ glycogen synthase kinase 3 beta (GSK3β)/β-Catenin signaling are analyzed by western blotting. Quantification of protein levels is carried out using ImageJ software version 1.53t.

Previous studies have reported that the dephosphorylation of β-Catenin at serine 33/37 and threonine 41 residues is essential for its nuclear translocation [70]. These phosphorylation sites induce the proteasomal degradation of β-Catenin and are primarily targeted by glycogen synthase kinase-3 beta (GSK3β), an upstream kinase [73]. GSK3β, a proline-directed serine/threonine kinase, regulates over 100 diverse proteins, including β-Catenin [72]. Its constitutive kinase activity is typically inhibited by upstream signals through specific phosphorylation events [73]. The Tyr216 residue in GSK3β is necessary for its activation, whereas Ser9, Thr43, and Thr390 are well-established inhibitory phosphorylation sites that induce its inhibition [74]. Therefore, we aimed to identify the upstream signaling pathways involved in β-Catenin activation by PG. As depicted in Fig. 4A, phosphorylation of β-Catenin at serine 33/37 and threonine 41 residues decreased, leading to increased stabilization of β-Catenin. Phosphorylation of GSK3β at the serine 9 residue was also upregulated, while the threonine 390 residue of phosphorylated GSK3β remained unchanged. The serine 9 residue of GSK3β is a well-known inhibitory site that hinders the function of GSK3β when phosphorylated [74].

**Fig. 5:**
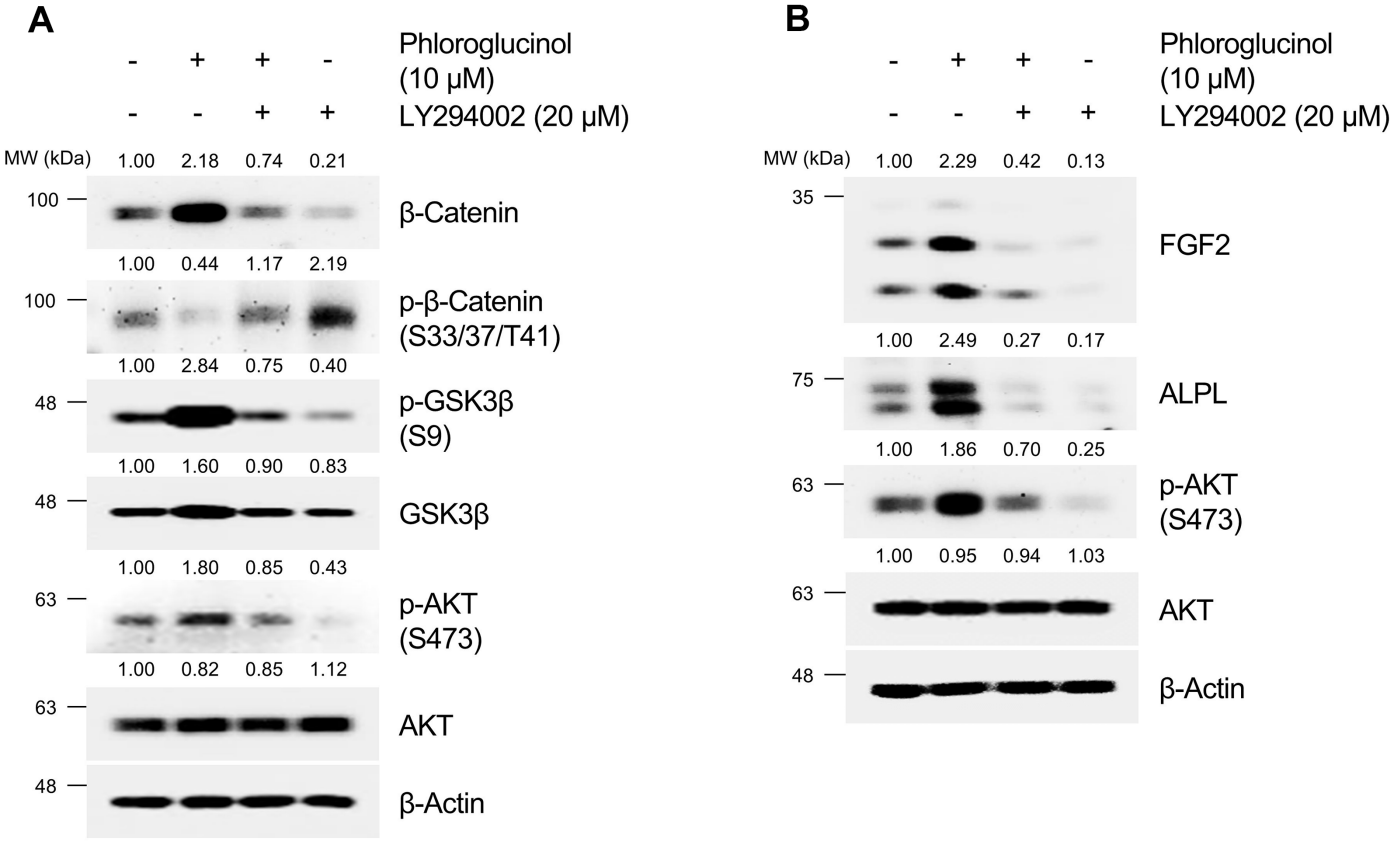
Effects of phloroglucinol (PG) on anagen signaling by activation of AKT/GSK3β/β-Catenin signaling pathway in HDPCs. (**A-B**) HDPCs are treated with 10 μM PG with or without 20 μM LY294002 for 12 h. Phosphorylation of AKT/GSK3β/β-Catenin signaling and anagen-inductive proteins fibroblast growth factor 2 (FGF2) and alkaline phosphatase (ALPL) are assessed by western blotting, with β-Actin as a loading control. Quantification of protein levels is carried out using ImageJ software version 1.53t.

Subsequently, we further identified the upstream kinases of GSK3β signaling regulated by PG. PG treatment did not affect the phosphorylation of protein kinase A (PKA), extracellular signal-activated kinase (ERK), and p38 (Fig. 4B). However, cells treated with 10 μM PG exhibited an increase in the phosphorylation level of AKT at the serine 473 residue, which targets the inhibitory phosphorylation site of GSK3β at serine 9 [75]. As expected, our results confirmed that PG (10 μM) increased the stabilization of β-Catenin, exhibiting a similar effect to the positive control, CHIR99021 (5 μM) (Fig. 4C). Additionally, PG (5 and 10 μM) activated the phosphorylation of AKT at serine 473 residue and GSK3β at serine 9 residue in a dose-dependent manner (Fig. 4C). We then investigated whether PG enhances anagen-inductive proteins and GSK3β/β-Catenin signaling through the activation of the AKT signaling pathway. For this purpose, we used the selective phosphoinositide 3-kinase (PI3K)/AKT inhibitor LY294002 [76]. As shown in Fig. 5A, co-treatment with PG (10 μM) and LY294002 (20 μM) abrogated the stabilization of β-Catenin. Cells co-treated with PG and LY294002 also showed a reduction in the phosphorylation levels of AKT at serine 473 residue and GSK3β at serine 9 residue (Fig. 5A). Moreover, it was demonstrated that co-treatment with PG and LY294002 alleviated the PG-induced increase in levels of anagen-inductive proteins (FGF2 and ALPL) (Fig. 5B). These findings demonstrate that PG-induced increase in anagen signaling and GSK3β/β-Catenin signaling is mediated through the activation of the AKT signaling pathway in HDPCs.

### Conditioned Medium from PG-Treated HDPCs Promotes Proliferation and Migration of Human Epidermal Keratinocytes

During the anagen phase of hair follicle, DPCs secrete paracrine factors, including vascular endothelial growth factor (VEGF), FGF2, FGF7, FGF10, insulin-like growth factor 1 (IGF1), and NOG, to promote the proliferation, migration, and differentiation of the surrounding follicular keratinocytes [77]. Recent studies have emphasized the indispensable role of increased β-Catenin signaling within DPCs in activating follicular keratinocytes and initiating the anagen phase [78]. Consequently, previous studies have utilized epithelial cell lineages such as hfSCs, HaCaT cells, and outer root sheath (ORS) cells to validate the hair growth-promoting properties of DPCs [79, 80]. Therefore, we investigated whether conditioned medium (CM) from PG-treated HDPCs stimulates the proliferation and migration of ORS-like HaCaT cells. As depicted in Fig. S1A, PG showed no cytotoxicity in HaCaT cells at concentrations up to 20 μM.

**Fig. 6:**
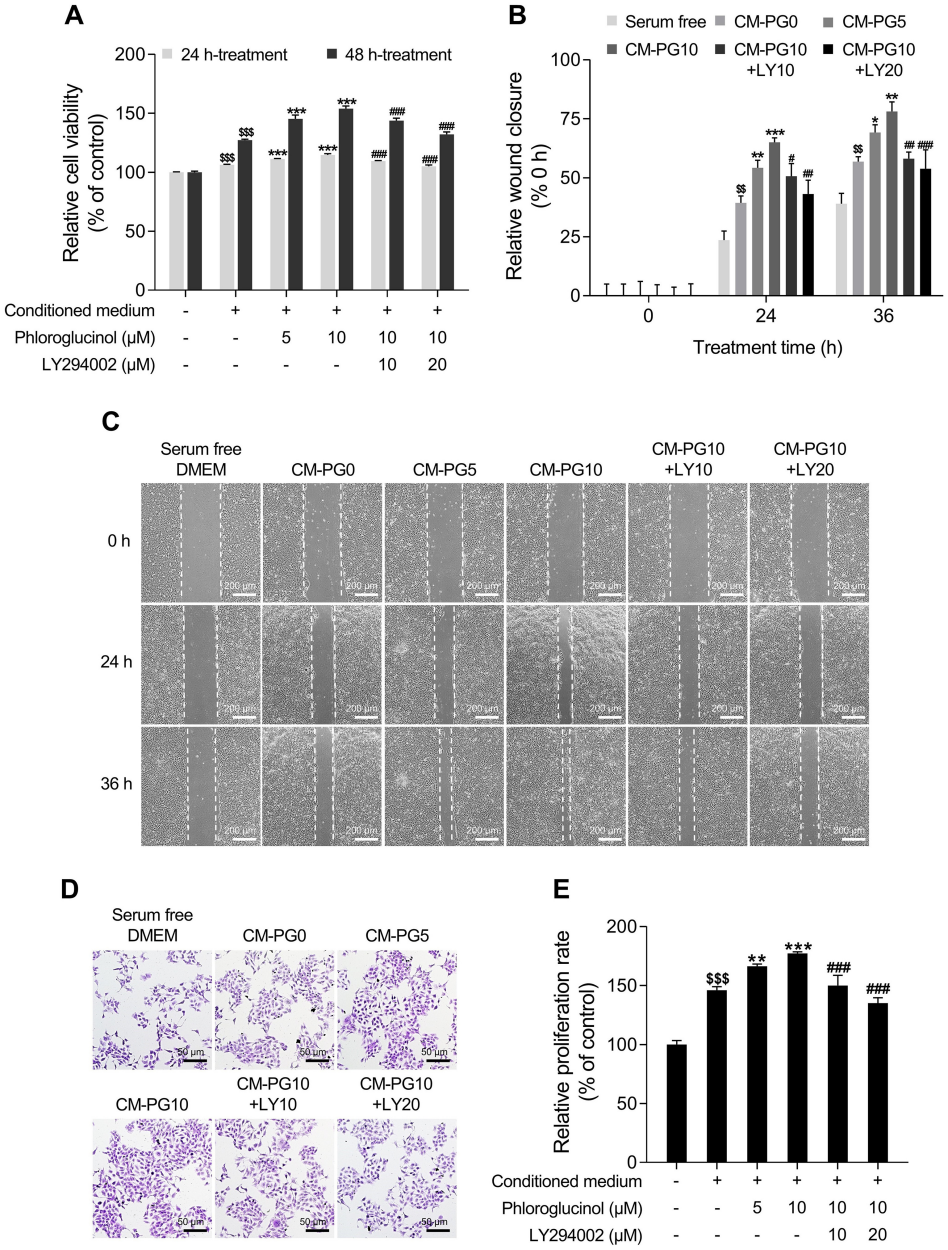
Effects of conditioned medium (CM) from phloroglucinol (PG)-treated HDPCs on the proliferation and migration efficacy of HaCaT cells. (**A**) HDPCs are treated with PG (5 or 10 μM) with or without LY294002 (10 or 20 μM) for 24 h. Then, the cells are replaced with serum-free DMEM, and further incubated for an additional 24 h to prepare CM. The indicated types of CM or serum free DMEM are applied to HaCaT cells for 48 h, and cell viabilities are assessed using a WST-1 assay. (**B**) HaCaT cells are treated with the indicated types of CM or serum free DMEM. The cells are then incubated for 36 h. Quantification of the wound closure rates is carried out using ImageJ software version 1.53t. (**C**) Representative microscopy images of the wound healing assays in the various medium are captured at 0 h, 24 h, and 36 h. (**D**) Images of the indicated types of CM or serum free DMEM treated HaCaT cells captured by phase-contrast microscopy following staining crystal violet solution. (**E**) Proliferation rates of the indicated types of CM or serum free DMEM treated HaCaT cells are measured via clonogenic assay. The results are presented as the mean ± SD and significant differences are analyzed using a one-way analysis of variance followed by Tukey’s test. ^$$^*p* < 0.01; ^$$$^*p* < 0.001 compared with the vehicle-treated group. **p < 0.01; ***p < 0.001 compared with the CM-PG0-treated group. ^#^*p* < 0.05; ^##^*p* < 0.01; ^###^*p* < 0.001 compared with the CM-PG10-treated group.

Subsequently, we examined whether PG-treated HDPCs stimulate the proliferation and migration of keratinocyte cell lineage by secreting various paracrine factors. For this purpose, we prepared CM from HDPCs and utilized it in subsequent experiments. WST-1 assay and clonogenic assay were employed to evaluate proliferation rate, while an in vitro wound healing assay was conducted to assess migration ability. As shown in Fig. 6A-6E, our results revealed that CM from PG-treated HDPCs (CM-PG0, CM-PG5, CM-PG10) significantly enhanced the proliferation and migration of HaCaT cells in a dose-dependent manner. Specifically, compared to CM-PG0, CM-PG5 and CM-PG10 increased proliferation rate of HaCaT cells by 20.30% and 31.36% respectively after 48 h treatment. These results were further observed by crystal violet staining under microscopy (Fig. 6D). The in vitro wound healing assay, also known as the scratch assay, was utilized to assess cell migration. Consequently, we confirmed the migratory capability of CM from PG-treated HDPCs. As shown in Fig. 6B, compared to the cells treated with CM-PG0 for 36 h, the cells treated with CM-PG5 and CM-PG10 showed increased closure of the wound area by 8.18% and 21.28%, respectively. These findings suggest that HDPCs induced upregulation of β-catenin and anagen signaling by PG may stimulate cellular expansion and movement of the keratinocyte cell lineage through various secretory factors.

The results of our study demonstrate that PG phosphorylates the Ser473 residue of AKT, thereby promoting anagen signaling in HDPCs (Fig. 5). Therefore, we aimed to determine whether the proliferation and migration-promoting effects of CM from PG-treated HDPCs on HaCaT cells are dependent on AKT signaling. Our results demonstrated that CM from HDPCs co-treated with PG and LY294002 (CM-PG10+LY10, CM-PG10+LY20) did not stimulate proliferation and migration of HaCaT cells compared to CM-PG10 (Fig. 6A-6E). Particularly, when cells were treated with CM-PG10+LY20 for 48 h, proliferation rate decreased by 42.15% compared to treatment with CM-PG10 alone (Fig. 6E). In the in vitro wound healing assay, treatment of HaCaT cells with CM-PG10+LY20 for 36 h resulted in a 24.26% decrease in wound closure area compared to treatment with CM-PG10 alone. Overall, our findings suggest that the proliferation and migration-promoting effects of CM from PG-treated HDPCs on keratinocytes may be induced by AKT signaling pathway.

### PG Exhibits Anti-Oxidative Abilities and Alleviates H_2_O_2_-Induced Cellular Stress

**Fig. 7:**
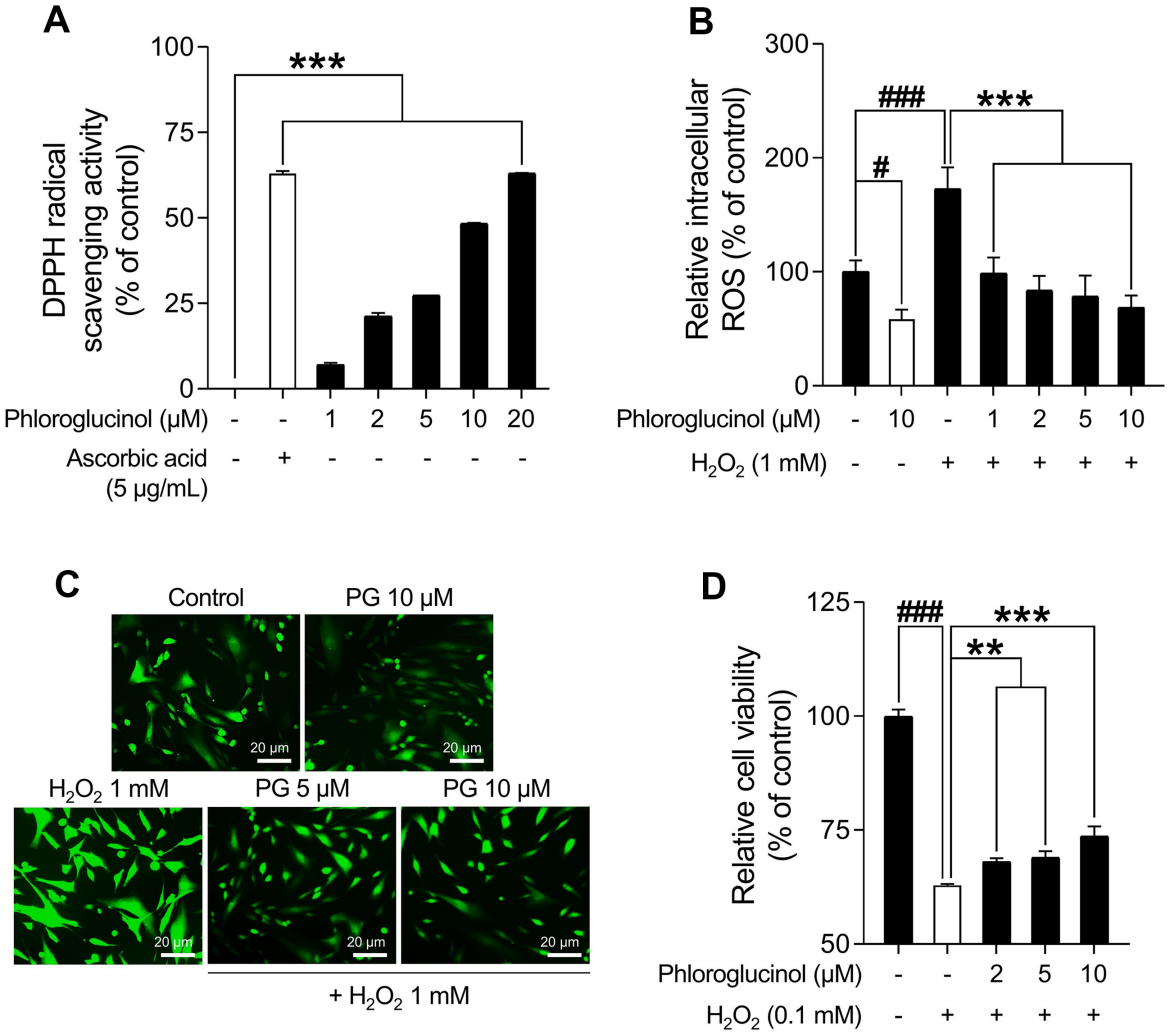
In vitro antioxidant activities of phloroglucinol (PG). (**A**) DPPH radical scavenging activity of PG. Ascorbic acid (5 μg/ml) is used as a positive control. (**B**) Intracellular reactive oxygen species (ROS) levels in HDPCs are assessed using a dichlorodihydrofluorescein diacetate (DCF-DA) microplate reader assay. (**C**) Representative images of DCF fluorescence are captured with an Axiovert 200 ultraviolet microscope. (**D**) The decreased toxicity of HDPCs treated with PG against H_2_O_2_- stimulation. HDPCs are exposed to indicated concentrations of PG (0-10 μM) for 30 min followed by treatment with 0.1 mM H_2_O_2_ for 48 h. The cell viabilities are assessed using a WST-1 assay. The results are presented as the mean ± SD of three independent experiments and are analyzed using a one-way analysis of variance followed by Tukey’s test. ^#^*p* < 0.05; ^###^*p* < 0.001 compared with the vehicle-treated group. **p < 0.01; ***p < 0.001 compared with the 0.1 mM H_2_O_2_-treated group.

**Fig. 8:**
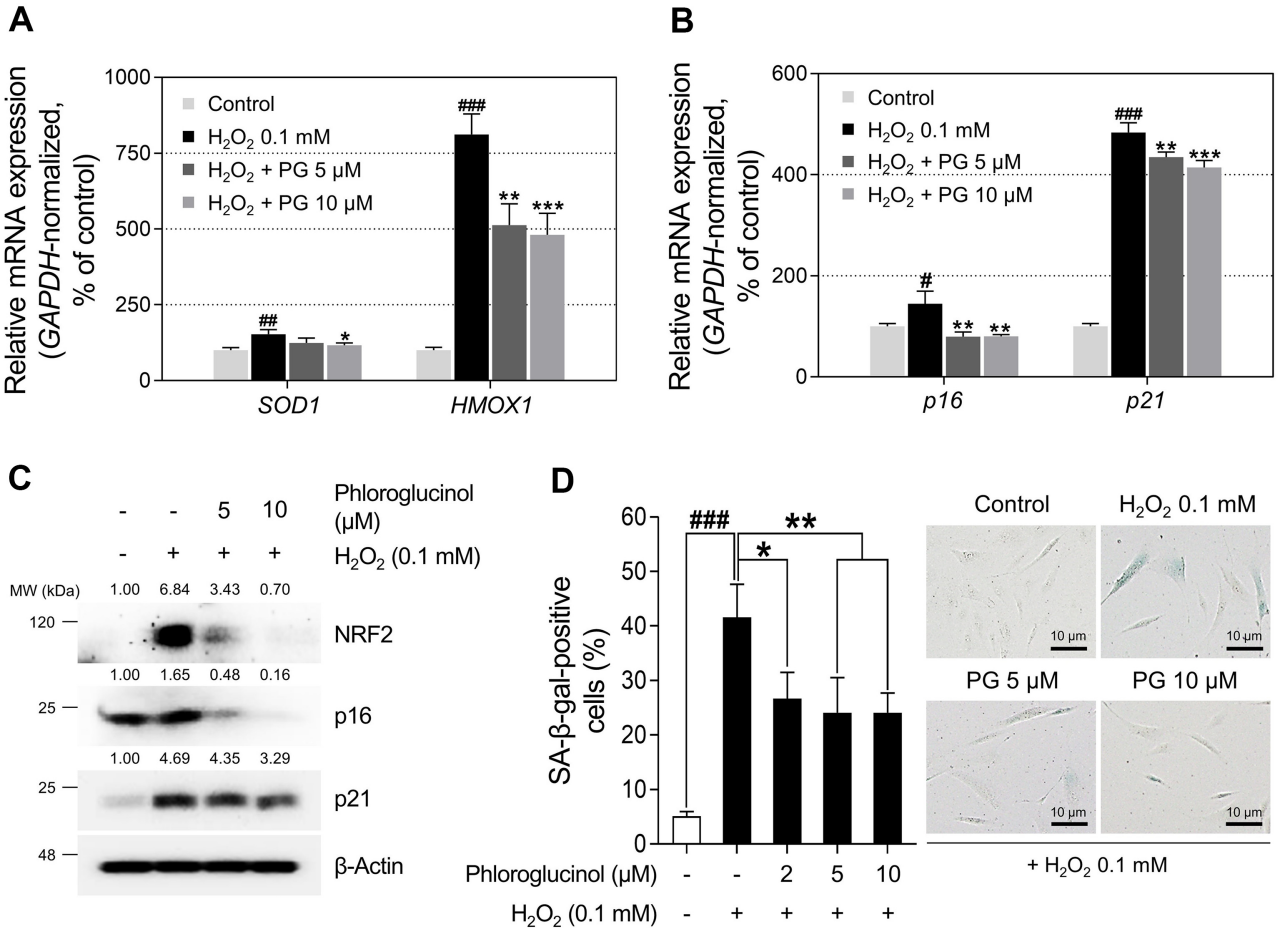
Protective effects of phloroglucinol (PG) on H_2_O_2_-induces cellular stress in HDPCs. (A-B) HDPCs are treated with PG (5 or 10 μM) with or without H_2_O_2_ (0.1 mM) for 24 h. The expression levels of oxidative stress markers (*HMOX1* and *SOD1*) and senescence-associated genes (*p16* and *p21*) are detected via qRT-PCR and normalized against *GAPDH*. (**C**) Protein levels of p16, p21, and NRF2 are analyzed by western blotting, with β-Actin serving as a loading control. Quantification of protein levels is carried out using ImageJ software version 1.53t. (**D**) HDPCs are co-treated with indicated doses of PG (0-10 μM) with or without H_2_O_2_ (0.1 mM) for 72 h. SA-β-gal positive cells were then observed by microscopy (scale bars, 10 μm). Representative images of senescence-associated beta-galactosidase (SA-β-gal) stained HDPCs are captured by phase-contrast microscopy. The results are presented as the mean ± SD and significant differences are analyzed using a one-way analysis of variance followed by Tukey’s test. ^#^*p* < 0.05; ^##^*p* < 0.01; ^###^*p* < 0.001 compared with the vehicle-treated group. *p < 0.05; **p < 0.01; ***p < 0.001 compared with the 0.1 mM H_2_O_2_-treated group.

PG has been reported to alleviate oxidative stress and reduce intracellular reactive oxygen species (ROS) levels [81]. Notably, due to the presence of phenolic hydroxyl groups, PG demonstrates significant anti-oxidant capacities [82]. Hence, we conducted an in vitro study to verify whether PG possesses anti-oxidative properties in HDPCs. Firstly, through the DPPH assay, we verified that PG effectively scavenges free radicals (Fig. 7A). Similarly, PG reduced significant intracellular ROS levels in HDPCs (Fig. 7B-7C). In particular, PG (10 μM) alone led to a significant reduction in cellular ROS levels by 41.68% compared to the untreated group (Fig. 7B-7C). Cells co-treated with PG (0-10 μM) and hydrogen peroxide (H_2_O_2_, 1 mM) exhibited a considerable decrease in the intensity of dichlorofluorescein (DCF) fluorescence compared to the H_2_O_2_-treated negative control (Fig. 7B-7C). Consistent with these findings, we observed an improvement in cell viability of up to 10.81% in HDPCs co-treated with PG (0-10 μM) and H_2_O_2_ (0.1 mM) compared to those treated with H_2_O_2_ alone for 48 h (Fig. 7D). These results suggest that PG may possess anti-oxidative properties within HDPCs, consistent with observations in other cell types. Given that PG mitigates oxidative stress and stress-induced premature senescence, we hypothesized that PG may alleviate H_2_O_2_-induced cellular stress in HDPCs [83]. Heme oxygenase-1 (HMOX1) and superoxide dismutase 1 (SOD1) are representative oxidative stress markers regulated by nuclear factor erythroid 2-related factor 2 (NRF2) [84, 85]. Particularly, ROS prevents proteasomal degradation of NRF2 through conformational changes of kelch-like ECH-associated protein 1 (KEAP1) and increases the expression of antioxidant response element (ARE)-dependent antioxidant target genes [86, 87]. In our results, H_2_O_2_ (0.1 mM) upregulated the mRNA levels of oxidative stress markers (*HMOX1* and *SOD1*) in HDPCs (Fig. 8A). Co-treatment with PG (0-10 μM) and H_2_O_2_ (0.1 mM) resulted in dose-dependent reductions in the mRNA levels of *HMOX1* and *SOD1* (Fig. 8A). Furthermore, the senescence-associated markers *p16* and *p21*, which were increased by H_2_O_2_, were alleviated by co-treatment with PG (Fig. 8B). H_2_O_2_ (0.1 mM) significantly elevated the protein levels of NRF2, p16, and p21 as a response to oxidative stress (Fig. 8C). In contrast, cells co-treated with PG (5 or 10 μM) and H_2_O_2_ (0.1 mM) exhibited a dose-dependent decrease in oxidative stress markers (NRF2) and senescence-associated proteins (p16 and p21) (Fig. 8C). Next, the anti-senescent effects of phloroglucinol in HDPCs were evaluated. PG significantly suppressed the intensity of senescence-associated beta-galactosidase (SA-β-gal) in H_2_O_2_-stimulated HDPCs (Fig. 8D). Notably, the number of blue-stained senescent cells decreased in a dose-dependent manner when co-treated with PG (0-10 μM) and H_2_O_2_ (0.1 mM). In the H_2_O_2_-treated negative control, the percentage of SA-β-gal positive senescent cells increased by 41.63% compared to untreated cells. In contrast, cells co-treated with H_2_O_2_ (0.1 mM) and 10 μM PG exhibited a 17.56% decrease compared to the negative control (Fig. 8D). Additionally, to validate the promising anti-hair loss properties of PG, we investigated its potential to mitigate cytotoxicity induced by DHT in HDPCs (Fig. S2). As shown in Fig. S2, PG exhibited a mild alleviation of cytotoxicity induced by DHT. These findings imply that PG might mitigate intracellular ROS induced by DHT, thus contributing to its protective effects (Fig. S2B). Hence, these results suggest that PG possesses in vitro anti-oxidative activities and alleviates oxidative stress-induced senescence within HDPCs.

### PG Reveals Protective Effects against the Attenuation of Anagen Signaling Induced by H_2_O_2_ in HDPCs

**Fig. 9:**
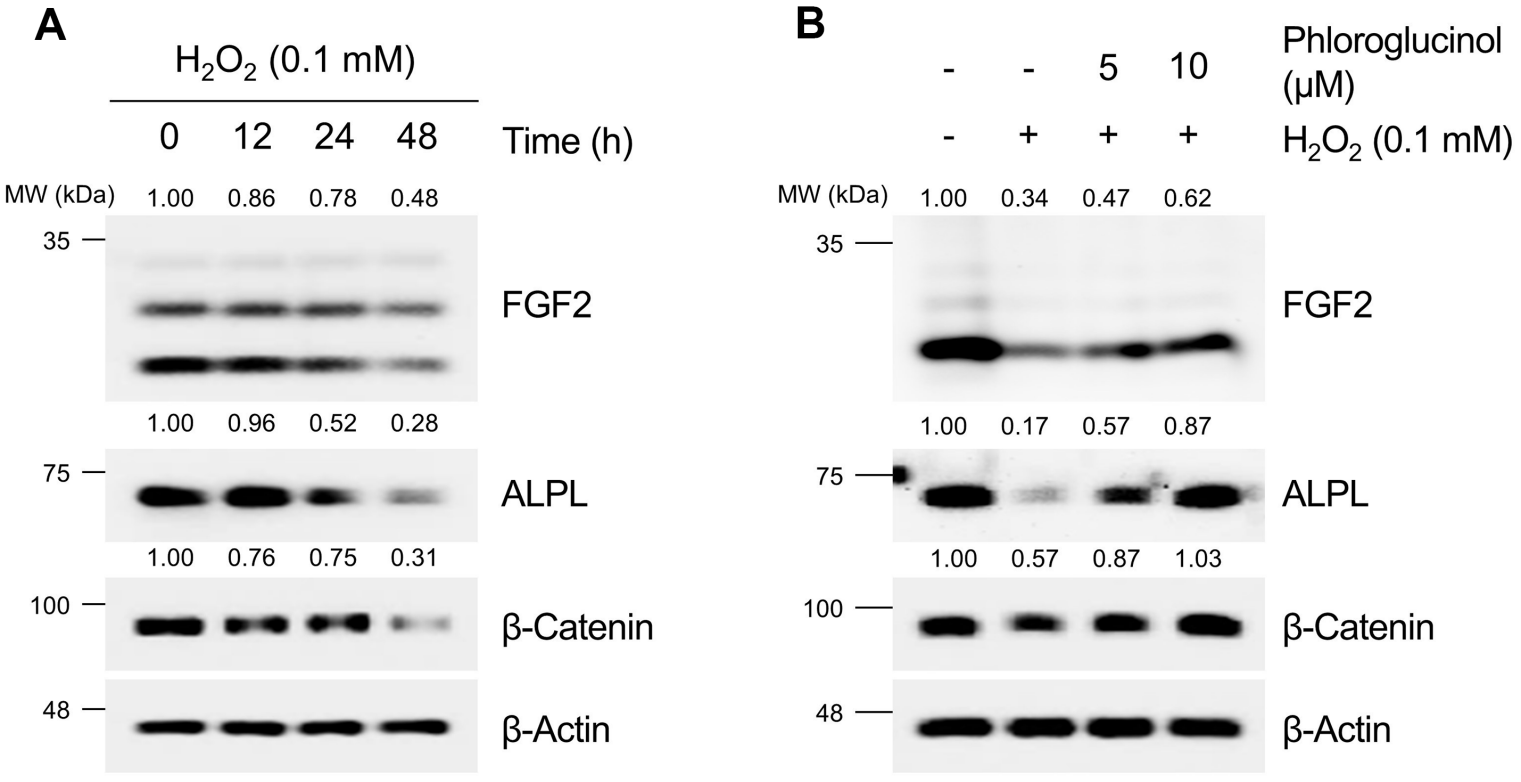
Protective effects of phloroglucinol (PG) against the attenuation of anagen signaling induced by H_2_O_2_ in HDPCs. (**A**) β-Catenin and anagen-inductive protein levels are examined at different time points after treatment with H_2_O_2_ (0.1 mM). (**B**) HDPCs are treated with PG (5 or 10 μM) with or without H_2_O_2_ (0.1 mM) for 48 h. The expression levels of anagen-inductive proteins (FGF2 and ALPL) and β-Catenin are analyzed by western blotting, with β-Actin serving as a loading control. Quantification of protein levels is carried out using ImageJ software version 1.53t.

Oxidative stress, recognized as one of the etiological factors of hair loss, has been reported to weaken β-Catenin signaling in HDPCs [40, 56]. Previous studies have indicated that the phosphorylation of the Ser9 residue of GSK3β is downregulated in H_2_O_2_-treated HDPCs, leading to decreased stability of β-Catenin [88]. Additionally, the enzyme activity of ALPL, a representative marker of anagen signaling, is reduced in H_2_O_2_-induced HDPCs [89]. Furthermore, CM from H_2_O_2_-treated HDPCs inhibits proliferation of keratinocytes, suggesting that excessive oxidative stress in HDPCs can attenuate anagen signaling [89]. In our results, PG significantly alleviated H_2_O_2_-induced oxidative stress in HDPCs. Therefore, we aimed to investigate whether PG prevents the weakening of β-Catenin and anagen signaling induced by H_2_O_2_ in HDPCs. As depicted in Fig. 9A, treatment with 0.1 mM H_2_O_2_ resulted in a time-dependent decrease in the protein levels of β-Catenin, FGF2, and ALPL. Subsequently, in further experiments, anagen signaling was assessed after treating cells with H_2_O_2_ for 48 h. As shown in Fig. 9B, cells treated with H_2_O_2_ alone exhibited a significant decrease in the expression levels of β-Catenin, ALPL, and FGF2 proteins. In contrast, cells co-treated with PG (0-10 μM) showed a dose-dependent restoration of the protein levels of β-catenin, ALPL, and FGF2 compared to cells treated with H_2_O_2_ alone. These findings suggest that PG has protective effects against the attenuation of anagen signaling induced by H_2_O_2_ in HDPCs.

## Discussion

The results of our study demonstrate that PG enhances anagen signaling by upregulating AKT/GSK3β/β-Catenin signaling, thereby stimulating keratinocyte proliferation and migration through the upregulation of secretory factors in HDPCs. Furthermore, PG protects against H_2_O_2_-induced oxidative stress and senescence, while preserving the reduced anagen signaling induced by H_2_O_2_ in HDPCs. These findings implicate PG as a potential therapeutic candidate for novel alopecia treatments by alleviating hair loss and enhancing hair growth.

The discovery of therapeutic candidates for hair loss treatment can be broadly categorized into two main strategies [90]. Firstly, there is an exploration of agents that promote hair growth. The intricate interplay between mesenchymal and epithelial cells orchestrates the regulation of hair growth, with HDPCs playing pivotal role [91]. Activation of Shh or β-Catenin signaling within HDPCs is critical for activating the epithelial cell lineage, particularly hfSCs, ultimately leading to hair follicle regeneration [50, 92]. As a result, numerous prior studies have employed in vitro models to assess the level of β-Catenin signaling within HDPCs, aiming to identify therapeutic candidates that induce hair growth [40, 76, 93]. The second approach involves shielding HDPCs from various etiological factors contributing to hair loss, notably oxidative stress and DHT [3]. DHT selectively acts on DPCs within the hair follicle, inducing senescence and promoting the secretion of WNT/β-Catenin signaling antagonists such as Dickkopf-1, thereby triggering hair regression [20, 94-96]. Oxidative stress also induces DPC senescence, promotes the secretion of inflammatory cytokines like IL-6, and inhibits anagen signaling within DPCs, contributing to hair regression [89]. Consequently, previous studies have sought to identify various therapeutic agents with protective effects against oxidative stress and hyper-androgenicity within HDPCs [23, 97-99]. Therefore, we aimed to explore the potential of PG, known to be abundant in brown algae, as a novel therapeutic agent for hair loss treatment and elucidate its mode of action within HDPCs. PG is a common secondary metabolite that is found in a free state or polymerized as phlorotannins in algae and is also present in higher plants [100]. It is one of the three isomeric benzene triols, containing a high content of monophenols, which has been widely exploited in pharmaceutical formulations [43]. In particular, plants (Myrtaceae family) and brown algae (*Ecklonia cava*) that contain PG and its derivatives have been traditionally used as medicinal substances for various treatments, including antimicrobial, anti-inflammatory, antioxidant, and hypoglycemic purposes [23]. Recent studies have reported that PG protects human keratinocytes from cell damage caused by ultraviolet B radiation and oxidative stress, increasing its potential use as an ingredient for skin improvement [24, 25]. Furthermore, PG derivatives, such as 7-phloroeckol and dieckol, have increased hair growth in vivo. The precise molecular mechanism of PG on hair growth in HDPCs has not been elucidated [3, 26]. This study aims to determine whether PG increases anagen-inductive properties in HDPCs and discover its molecular mechanisms.

In our results, PG stimulated the expression of anagen-inductive genes without causing cytotoxicity. Previous studies have demonstrated that the activation of the WNT/β-Catenin signaling pathway in DPCs is crucial for hair growth [50, 101]. Therefore, to determine the impact of PG on the β-Catenin signaling pathway and its effect on anagen-inductive genes in HDPCs, we performed various biochemical analyses, along with TCF/LEF luciferase reporter assays and nuclear fractionation. Our findings demonstrated that PG increased transcriptional activities of β-Catenin, similar to lithium chloride (5 mM) and CHIR99021 (5 μM), which were used as positive controls. Since PG did not increase the mRNA expression of *WNT* in HDPCs, it was hypothesized that it may affect proteostasis of β-Catenin. The protein stability of the transcriptional factor β-Catenin is primarily regulated by ubiquitin-mediated proteasomal degradation [69]. In our study, we found that PG modulates β-Catenin signaling by increasing protein stability rather than enhancing gene expression. Furthermore, through additional experiments using the proteasome inhibitor MG132, we demonstrated that PG activates the β-Catenin signaling pathway by suppressing proteasome-mediated β-Catenin degradation.

GSK3β, a proline-directed serine/threonine kinase, regulates a wide array of proteins, including β-Catenin [72]. GSK3β exhibits constitutive kinase activity, which is regulated by activation at the Tyr216 residue [70]. Conversely, activation of residues Ser9, Thr43, and Thr390 of GSK3β inhibits its kinase activity, thereby attenuating ubiquitin-mediated proteolysis of β-Catenin [69, 70, 73]. In our findings, PG increased the phosphorylation levels of β-Catenin at serine 33/37 and threonine 41 residues, resulting in higher protein stability of β-Catenin. PG increased the phosphorylation level of GSK3β at serine 9 residue, but it did not affect the threonine 390 residue. Furthermore, PG showed no impact on upstream kinases of GSK3β, such as PKA, p38, and ERK, but it increased the phosphorylation level of serine 473 residue on AKT. We confirmed that PG increased AKT/GSK3β/β-Catenin signaling in a dose-dependent manner. Considering these results, we investigated whether PG's enhancement of GSK3β/β-Catenin signaling is mediated by the AKT signaling pathway. Our results demonstrated that LY294002, a selective PI3K/AKT inhibitor, could reverse the increased AKT/GSK3β/β-Catenin signaling induced by PG. These results are consistent with previous findings, indicating that the phosphorylation of AKT at serine 473 residue reduces the activity of GSK3β and induces stabilization of β-Catenin [76, 102-104]. Moreover, when AKT signaling was suppressed, the expression levels of anagen-inductive proteins increased by PG (FGF2 and ALPL) were restored to levels similar to the untreated control. Conditioned medium from PG-treated HDPCs stimulated the proliferation and migration of human epidermal keratinocytes. The production of beneficial secretory factors derived from the CM of PG-treated HDPCs suggests the potential engagement of the AKT/GSK3β/β-Catenin pathway. These findings demonstrate that PG enhances the signal transduction of GSK3β/β-Catenin by activating AKT signaling, thereby strengthening anagen signaling.

Recent studies have suggested that oxidative stress may be an etiological factor in hair loss [105-109]. The expression of oxidative stress markers in the blood of patients has been observed to increase [109, 110]. Additionally, the HDPCs of individuals with pattern baldness are sensitive to oxidative stress and the hair follicles treated with H_2_O_2_ undergo the catagen phase [88, 107, 111]. Therefore, we investigated whether PG has anti-oxidative abilities in HDPCs. Consistent with previous studies, our data showed that PG reduced ROS levels in HDPCs [35, 112]. Additionally, when HDPCs were exposed to H_2_O_2_ and then treated with PG, both oxidative stress markers (*HMOX1* and *SOD1*) and senescence markers (*p16* and *p21*) were significantly alleviated. This led us to hypothesize that PG mitigates oxidative stress by decreasing p16 and p21 protein levels. Experimentally, we observed a dose-dependent reduction in SA-β-gal-positive senescent cells treated with PG. PG also demonstrated protective effects against the reduction of β-Catenin and anagen signaling caused by oxidative stress within HDPCs, confirming its protective role against oxidative stress. Furthermore, PG mildly improved cytotoxicity induced under hyper-androgenic conditions, potentially by inhibiting intracellular ROS generated by DHT. Overall, our data suggest that PG possesses intracellular antioxidant activities, offering a potential therapeutic avenue for alleviating hair loss.

In the present study, we found the anagen-promoting effects of PG within the in vitro model, confirming its potential to induce hair growth and elucidating its molecular mechanism. Furthermore, PG exhibited anti-oxidative activities and mitigated senescence in HDPCs induced with oxidative stress, a known etiological factor of alopecia.

In conclusion, PG significantly enhanced anagen signaling through the AKT/GSK3β/β-Catenin signaling pathway and alleviated cellular damage caused by oxidative stress. Although our findings provide valuable insights based on HDPCs, further research is required to validate these effects within the entire human hair follicle system.

## Supplemental Materials

Supplementary data for this paper are available on-line only at http://jmb.or.kr.



## Figures and Tables

**Table 1:** List of sequences used for qRT-PCR.

Target mRNA	Sequences of primer	Annealing temperature (℃)
*ALPL* (Alkaline phosphatase)	F : 5’-CAAACCGAGATACAAGCACTCCC-3’	58
	R : 5’-CGAAGAGACCCAATAGGTAGTCCAC-3’	
*FGF7* (Fibroblast growth factor 7)	F : 5’-GCATATTGAGGGCAGAGGAGGAC-3’	58
	R : 5’-GGATGGAGGCCCCTTACAGTTT-3’	
*FGF10* (Fibroblast growth factor 10)	F : 5’-CACATTGTGCCTCAGCCTTTC-3’	58
	R : 5’-AGGTGATTGTAGCTCCGCACA-3’	
*WNT1* (Wnt family member 1)	F : 5’-AGGAGGTGAGAGAAGGATGGGT-3’	58
	R : 5’-CATTTCTGCTGGTTCCCCCAAC-3’	
*WNT3A* (Wnt family member 3)	F : 5’-CCTGGAGCTAGTGTCTCCTCTCT-3’	58
	R : 5’-CAGGAAGAAGCCTCATCCACCAT-3’	
*WNT4* (Wnt family member 4)	F : 5’-GAGGAGACGTGCGAGAAACTCAA-3’	58
	R : 5’-ATCCTGACCACTGGAAGCCCTGT-3’	
*WNT7B* (Wnt family member 7B)	F : 5’-AGCCCTGTCCTGGTCCTTTTAC-3’	58
	R : 5’-CCCTCTGTCACTCATGCTCCTC-3’	
*WNT10A* (Wnt family member 10A)	F : 5’-TGCACCGCTTACAACTGGAT-3’	58
	R : 5’-TTCTCGCGTGGATGTCTCTG-3’	
*WNT10B* (Wnt family member 10B)	F : CCTGAAGCGGAAATGCAAGTGT	58
	R : GGCTGACCCTCACTTACACACA	
*CTNNB1* (Catenin beta 1)	F : 5’-AAAATGGCAGTGCGT TTAG-3’	52
	R : 5’-TTTGAAGGCACTCTGTCGTA-3’	
*p16* (Cyclin dependent kinase inhibitor 2A)	F : 5’-TGCCTTTTCACTGTGTTGGA-3’	55
	R : 5’-GCCATTTGCTAGCAGTGTGA-3’	
*p21* (Cyclin dependent kinase inhibitor 1A)	F : 5’-GAACTTCGACTTTGTCACCGAGAC-3’	56
	R : 5’-TGGAGTGGTAGAAATCTGTCATGCT-3’	
*SOD1* (Superoxide dismutase 1)	F : 5’-CCAGTGCAGGGCATCATCA-3’	56
	R : 5’-TTGGCCCACCGTGTTTTCT-3’	
*HMOX1* (Heme oxygenase 1)	F : 5’-GCCCTTCAGCATCCTCAGTTCC-3’	60
	R : 5’-AGTGGTCATGGCCGTGTCAAC-3’	
*GAPDH* (Glyceraldehyde-3-phosphate dehydrogenase)	F : 5’-TCCAAAATCAAGTGGGGCGATGC-3’	60
	R : 5’-GCCAGTAGAGGCAGGGATGATGT-3’

**Table 2:** List of antibodies for western blot analyses.

Antigen	Host	Clonality (Species reactivity)	Dilution	Manufacturer (Cat. number)
β-Catenin	Rabbit	Polyclonal (Human, Mouse, Rat, Monkey)	1:1000	Cell Signaling Technology (#9562)
Lamin A/C	Mouse	Monoclonal (Human, Mouse, Rat, Monkey)	1:1000	Cell Signaling Technology (#4777)
α-Tubulin	Mouse	Monoclonal (Human, Mouse, Rat, Monkey)	1:1000	Cell Signaling Technology (#3873)
p-β-Catenin (S33/37/T41)	Rabbit	Polyclonal (Human, Mouse, Rat, Monkey)	1:1000	Cell Signaling Technology (#9561)
GSK3β	Rabbit	Monoclonal (Human, Mouse, Rat, Monkey)	1:1000	Cell Signaling Technology (#9315)
p-GSK3β (S9)	Rabbit	Monoclonal (Human, Mouse, Rat, Monkey)	1:1000	Cell Signaling Technology (#9323)
p-GSK3β (T390)	Rabbit	Polyclonal (Human)	1:1000	Cell Signaling Technology (#3548)
AKT	Rabbit	Polyclonal (Human, Mouse, Rat, Monkey•••)	1:1000	Cell Signaling Technology (#9272)
p-AKT (S473)	Rabbit	Polyclonal (Human, Mouse, Rat, Monkey•••)	1:1000	Cell Signaling Technology (#9271)
PKA	Rabbit	Polyclonal (Human, Mouse, Rat)	1:1000	Cell Signaling Technology (#4782)
p-PKA (T197)	Rabbit	Polyclonal (Human, Mouse, Rat, Monkey)	1:1000	Cell Signaling Technology (#4781)
p38	Rabbit	Monoclonal (Human, Mouse, Rat, Monkey•••)	1:1000	Cell Signaling Technology (#54470)
p-p38 (T180/Y182)	Rabbit	Polyclonal (Human, Mouse, Rat, Monkey•••)	1:1000	Cell Signaling Technology (#9211)
ERK	Rabbit	Polyclonal (Human, Mouse, Rat, Monkey•••)	1:1000	Cell Signaling Technology (#9102)
p-ERK (T202/Y204)	Rabbit	Polyclonal (Human, Mouse, Rat, Monkey•••)	1:1000	Cell Signaling Technology (#9101)
FGF2	Mouse	Monoclonal (Human, Mouse, Rat)	1:1000	Santa Cruz (#sc-365106)
ALPL	Mouse	Monoclonal (Human, Mouse, Rat)	1:1000	Santa Cruz (#sc-365765)
p16	Rabbit	Monoclonal (Human)	1:1000	Abcam (#ab108349)
p21	Rabbit	Monoclonal (Human, Monkey)	1:1000	Cell Signaling Technology (#2947)
β-Actin	Mouse	Monoclonal (Human, Mouse, Rat•••)	1:1000	Santa Cruz (#sc-47778)

## References

[1] Houschyar KS, Borrelli MR, Tapking C, Popp D, Puladi B, Ooms M (2020). Molecular mechanisms of hair growth and regeneration: current understanding and novel paradigms. Dermatology.

[2] Choi BY (2018). Hair-growth potential of ginseng and its major metabolites: a review on its molecular mechanisms. Int. J. Mol. Sci..

[3] Madaan A, Verma R, Singh AT, Jaggi M (2018). Review of hair follicle dermal papilla cells as in vitro screening model for hair growth. Int. J. Cosmet. Sci..

[4] Ji S, Zhu Z, Sun X, Fu X (2021). Functional hair follicle regeneration: an updated review. Signal Transduct. Target. Ther..

[5] Nilforoushzadeh M, Rahimi Jameh E, Jaffary F, Abolhasani E, Keshtmand G, Zarkob H (2017). Hair follicle generation by injections of adult human follicular epithelial and dermal papilla cells into nude mice. Cell J..

[6] Lin WH, Xiang LJ, Shi HX, Zhang J, Jiang LP, Cai PT (2015). Fibroblast growth factors stimulate hair growth through betacatenin and Shh expression in C57BL/6 mice. Biomed Res. Int..

[7] Kinoshita-Ise M, Tsukashima A, Kinoshita T, Yamazaki Y, Ohyama M (2020). Altered FGF expression profile in human scalpderived fibroblasts upon WNT activation: implication of their role to provide folliculogenetic microenvironment. Inflamm. Regen..

[8] Rishikaysh P, Dev K, Diaz D, Qureshi WM, Filip S, Mokry J (2014). Signaling involved in hair follicle morphogenesis and development. Int. J. Mol. Sci..

[9] Shin DW (2022). The molecular mechanism of natural products activating Wnt/beta-catenin signaling pathway for improving hair loss. Life (Basel).

[10] Lin BJ, Zhu JY, Ye J, Lu SD, Liao MD, Meng XC (2020). LncRNA-XIST promotes dermal papilla induced hair follicle regeneration by targeting miR-424 to activate hedgehog signaling. Cell. Signal..

[11] Hwang SB, Park HJ, Lee BH (2022). Hair-growth-promoting effects of the fish collagen peptide in human dermal papilla cells and C57BL/6 mice modulating Wnt/beta-catenin and BMP signaling pathways. Int. J. Mol. Sci..

[12] Cotsarelis G, Millar SE (2001). Towards a molecular understanding of hair loss and its treatment. Trends Mol Med..

[13] Williamson D, Gonzalez M, Finlay AY (2001). The effect of hair loss on quality of life. J. Eur. Acad. Dermatol. Venereol..

[14] Rushton DH (2002). Nutritional factors and hair loss. Clin. Exp. Dermatol..

[15] Brajac I, Tkalcic M, Dragojevic DM, Gruber F (2003). Roles of stress, stress perception and trait-anxiety in the onset and course of alopecia areata. J. Dermatol..

[16] McElwee KJ, Gilhar A, Tobin DJ, Ramot Y, Sundberg JP, Nakamura M (2013). What causes alopecia areata?. Exp. Dermatol..

[17] Kaufman KD (2002). Androgens and alopecia. Mol. Cell. Endocrinol..

[18] Magro CM, Rossi A, Poe J, Manhas-Bhutani S, Sadick N (2011). The role of inflammation and immunity in the pathogenesis of androgenetic alopecia. J. Drugs Dermatol..

[19] Katsarou-Katsari A, Singh LK, Theoharides TC (2001). Alopecia areata and affected skin CRH receptor upregulation induced by acute emotional stress. Dermatology.

[20] Bahta AW, Farjo N, Farjo B, Philpott MP (2008). Premature senescence of balding dermal papilla cells in vitro is associated with p16(INK4a) expression. J. Invest. Dermatol..

[21] Deng Y, Wang M, He Y, Liu F, Chen L, Xiong X (2023). Cellular senescence: ageing and androgenetic alopecia. Dermatology.

[22] Wang Y, Sui Y, Lian A, Han X, Liu F, Zuo K (2021). PBX1 attenuates hair follicle-derived mesenchymal stem cell senescence and apoptosis by alleviating reactive oxygen species-mediated DNA damage instead of enhancing DNA damage repair. Front. Cell. Dev. Biol..

[23] Jung YH, Chae CW, Choi GE, Shin HC, Lim JR, Chang HS (2022). Cyanidin 3-O-arabinoside suppresses DHT-induced dermal papilla cell senescence by modulating p38-dependent ER-mitochondria contacts. J. Biomed. Sci..

[24] Shin JY, Kim J, Choi YH, Kang NG, Lee S (2021). Dexpanthenol promotes cell growth by preventing cell senescence and apoptosis in cultured human hair follicle cells. Curr. Issues Mol. Biol..

[25] Devjani S, Ezemma O, Kelley KJ, Stratton E, Senna M (2023). Androgenetic alopecia: therapy update. Drugs.

[26] Suchonwanit P, Thammarucha S, Leerunyakul K (2019). Minoxidil and its use in hair disorders: a review. Drug Des. Devel. Ther..

[27] Gupta AK, Venkataraman M, Talukder M, Bamimore MA (2022). Finasteride for hair loss: a review. J. Dermatolog. Treat..

[28] Cao JQ, Huang XJ, Li YT, Wang Y, Wang L, Jiang RW (2016). Callistrilones A and B, triketone-phloroglucinol-monoterpene hybrids with a new skeleton from *Callistemon rigidus*. Org. Lett..

[29] Daus M, Wunnoo S, Voravuthikunchai SP, Saithong S, Poldorn P, Jungsuttiwong S (2022). Phloroglucinol-meroterpenoids from the leaves of *Eucalyptus camaldulensis* Dehnh. Phytochemistry.

[30] Ding XY, Wen JR, Lin WY, Huang GY, Feng Q, Duan L (2023). Phloroglucinol derivatives, coumarins and an alkaloid from the roots of *Evodia lepta* Merr. Phytochemistry.

[31] Daikonya A, Katsuki S, Wu JB, Kitanaka S (2002). Anti-allergic agents from natural sources (4): anti-allergic activity of new phloroglucinol derivatives from Mallotus philippensis (Euphorbiaceae). Chem. Pharm. Bull (Tokyo).

[32] Khan F, Tabassum N, Bamunuarachchi NI, Kim YM (2022). Phloroglucinol and its derivatives: antimicrobial properties toward microbial pathogens. J. Agric. Food Chem..

[33] Marasinghe CK, Jung WK, Je JY (2023). Phloroglucinol possesses anti-inflammatory activities by regulating AMPK/Nrf2/HO-1 signaling pathway in LPS-stimulated RAW264.7 murine macrophages. Immunopharmacol. Immunotoxicol..

[34] Zhang YB, Li W, Jiang L, Yang L, Chen NH, Wu ZN (2018). Cytotoxic and anti-inflammatory active phloroglucinol derivatives from *Rhodomyrtus tomentosa*. Phytochemistry.

[35] Park C, Cha HJ, Hong SH, Kim GY, Kim S, Kim HS (2019). Protective effect of phloroglucinol on oxidative stress-induced DNA damage and apoptosis through activation of the Nrf2/HO-1 signaling pathway in HaCaT human keratinocytes. Mar. Drugs.

[36] Piao MJ, Kim KC, Kang KA, Fernando P, Herath H, Hyun JW (2021). Phloroglucinol attenuates ultraviolet B-induced 8-oxoguanine formation in human HaCaT keratinocytes through Akt and Erk-mediated Nrf2/Ogg1 signaling pathways. Biomol. Ther..

[37] Piao MJ, Ahn MJ, Kang KA, Kim KC, Zheng J, Yao CW (2014). Phloroglucinol inhibits ultraviolet B radiation-induced oxidative stress in the mouse skin. Int. J. Radiat. Biol..

[38] Kim KC, Piao MJ, Cho SJ, Lee NH, Hyun JW (2012). Phloroglucinol protects human keratinocytes from ultraviolet B radiation by attenuating oxidative stress. Photodermatol. Photoimmunol. Photomed..

[39] Ng NS, Ooi L (2021). A simple microplate assay for reactive oxygen species generation and rapid cellular protein normalization. Bio Protoc..

[40] Park S, Han N, Lee JM, Lee JH, Bae S (2023). Effects of *Allium hookeri* extracts on hair-inductive and anti-oxidative properties in human dermal papilla cells. Plants (Basel, Switzerland)..

[41] Debacq-Chainiaux F, Erusalimsky JD, Campisi J, Toussaint O (2009). Protocols to detect senescence-associated beta-galactosidase (SA-betagal) activity, a biomarker of senescent cells in culture and in vivo. Nat. Protoc..

[42] Blackman AJ, Rogers GI, Volkman JK (1988). Phloroglucinol derivatives from three Australian marine algae of the genus Zonaria. J. Nat. Prod..

[43] Singh IP, Sidana J, Bansal P, Foley WJ (2009). Phloroglucinol compounds of therapeutic interest: global patent and technology status. Exp. Opin. Ther. Pat..

[44] Bak SS, Sung YK, Kim SK (2014). 7-Phloroeckol promotes hair growth on human follicles in vitro. Naunyn Schmiedebergs Arch. Pharmacol..

[45] Kang JI, Kim SC, Kim MK, Boo HJ, Jeon YJ, Koh YS (2012). Effect of dieckol, a component of *Ecklonia cava*, on the promotion of hair growth. Int. J. Mol. Sci..

[46] Iida M, Ihara S, Matsuzaki T (2007). Hair cycle-dependent changes of alkaline phosphatase activity in the mesenchyme and epithelium in mouse vibrissal follicles. Dev. Growth Differ..

[47] du Cros DL, LeBaron RG, Couchman JR (1995). Association of versican with dermal matrices and its potential role in hair follicle development and cycling. J. Invest. Dermatol..

[48] Suzuki K, Yamanishi K, Mori O, Kamikawa M, Andersen B, Kato S (2000). Defective terminal differentiation and hypoplasia of the epidermis in mice lacking the Fgf10 gene. FEBS Lett..

[49] Rosenquist TA, Martin GR (1996). Fibroblast growth factor signalling in the hair growth cycle: expression of the fibroblast growth factor receptor and ligand genes in the murine hair follicle. Dev. Dyn..

[50] Enshell-Seijffers D, Lindon C, Kashiwagi M, Morgan BA (2010). Beta-catenin activity in the dermal papilla regulates morphogenesis and regeneration of hair. Dev. Cell.

[51] Zhou L, Yang K, Xu M, Andl T, Millar SE, Boyce S (2016). Activating β-catenin signaling in CD133-positive dermal papilla cells increases hair inductivity. FEBS J..

[52] Kiso M, Hamazaki TS, Itoh M, Kikuchi S, Nakagawa H, Okochi H (2015). Synergistic effect of PDGF and FGF2 for cell proliferation and hair inductive activity in murine vibrissal dermal papilla in vitro. J. Dermatol. Sci..

[53] Liu J, Xiao Q, Xiao J, Niu C, Li Y, Zhang X (2022). Wnt/β-catenin signalling: function, biological mechanisms, and therapeutic opportunities. Signal Transduct. Target. Ther..

[54] Rao TP, Kühl M (2010). An updated overview on Wnt signaling pathways: a prelude for more. Circ. Res..

[55] Nie X, Liu H, Liu L, Wang YD, Chen WD (2020). Emerging roles of Wnt ligands in human colorectal cancer. Front. Oncol..

[56] Choi BY (2020). Targeting Wnt/β-catenin pathway for developing therapies for hair loss. Int. J. Mol. Sci..

[57] Dong L, Hao H, Xia L, Liu J, Ti D, Tong C (2014). Treatment of MSCs with Wnt1a-conditioned medium activates DP cells and promotes hair follicle regrowth. Sci. Rep..

[58] Kishimoto J, Burgeson RE, Morgan BA (2000). Wnt signaling maintains the hair-inducing activity of the dermal papilla. Genes Dev..

[59] Kandyba E, Kobielak K (2014). Wnt7b is an important intrinsic regulator of hair follicle stem cell homeostasis and hair follicle cycling. Stem Cells.

[60] Wu Z, Zhu Y, Liu H, Liu G, Li F (2020). Wnt10b promotes hair follicles growth and dermal papilla cells proliferation via Wnt/β-catenin signaling pathway in Rex rabbits. Biosci. Rep..

[61] Choi BY (2020). Targeting Wnt/β-catenin pathway for developing therapies for hair loss. Int. J. Mol. Sci..

[62] Lu GQ, Wu ZB, Chu XY, Bi ZG, Fan WX (2016). An investigation of crosstalk between Wnt/β-catenin and transforming growth factor-β signaling in androgenetic alopecia. Medicine.

[63] Werner J, Boonekamp KE, Zhan T, Boutros M (2023). The roles of secreted Wnt ligands in cancer. Int. J. Mol. Sci..

[64] Koch S (2021). Regulation of Wnt signaling by FOX transcription factors in cancer. Cancers.

[65] Park HB, Kim JW, Baek KH (2020). Regulation of Wnt signaling through ubiquitination and deubiquitination in cancers. Int. J. Mol. Sci..

[66] Rim EY, Clevers H, Nusse R (2022). The Wnt pathway: From signaling mechanisms to synthetic modulators. Ann. Rev. Biochem..

[67] Tian Q, Jin H, Cui Y, Guo C, Lu X (2005). Regulation of Wnt gene expression. Dev. Growth Differ..

[68] Liu J, Xiao Q, Xiao J, Niu C, Li Y, Zhang X (2022). Wnt/β-catenin signalling: function, biological mechanisms, and therapeutic opportunities. Signal Transduct. Target. Ther..

[69] Gao C, Xiao G, Hu J (2014). Regulation of Wnt/β-catenin signaling by posttranslational modifications. Cell Biosci..

[70] Liu C, Li Y, Semenov M, Han C, Baeg G-H, Tan Y (2002). Control of β-catenin phosphorylation/degradation by a dual-kinase mechanism. Cell.

[71] Zhao L, Zhao J, Zhong K, Tong A, Jia D (2022). Targeted protein degradation: mechanisms, strategies and application. Signal Transduct. Target. Ther..

[72] White KA, Grillo-Hill BK, Esquivel M, Peralta J, Bui VN, Chire I (2018). β-Catenin is a pH sensor with decreased stability at higher intracellular pH. J. Cell Biol..

[73] Liu C, Kato Y, Zhang Z, Do VM, Yankner BA, He X (1999). beta-Trcp couples beta-catenin phosphorylation-degradation and regulates Xenopus axis formation. Proc. Natl. Acad. Sci. USA.

[74] Fang X, Yu SX, Lu Y, Bast RC, Woodgett JR, Mills GB (2000). Phosphorylation and inactivation of glycogen synthase kinase 3 by protein kinase A. Proc. Natl. Acad. Sci. USA.

[75] Zhou X, Wang H, Burg MB, Ferraris JD (2013). Inhibitory phosphorylation of GSK-3β by AKT, PKA, and PI3K contributes to high NaCl-induced activation of the transcription factor NFAT5 (TonEBP/OREBP). Am. J. Physiol. Renal Physiol..

[76] Bae S, Yoon YG, Kim JY, Park IC, An S, Lee JH (2022). Melatonin increases growth properties in human dermal papilla spheroids by activating AKT/GSK3β/β-catenin signaling pathway. PeerJ..

[77] Driskell RR, Clavel C, Rendl M, Watt FM (2011). Hair follicle dermal papilla cells at a glance. J. Cell Sci..

[78] Morgan BA (2014). The dermal papilla: an instructive niche for epithelial stem and progenitor cells in development and regeneration of the hair follicle. Cold Spring Harbor Perspect. Med..

[79] Roh C, Tao Q, Lyle S (2004). Dermal papilla-induced hair differentiation of adult epithelial stem cells from human skin. Physiol. Genomics.

[80] Lee YR, Bae S, Kim JY, Lee J, Cho DH, Kim HS (2019). Monoterpenoid loliolide regulates hair follicle inductivity of human dermal papilla cells by activating the Akt/beta-catenin signaling pathway. J. Microbiol. Biotechnol..

[81] Quéguineur B, Goya L, Ramos S, Martín MA, Mateos R, Bravo L (2012). Phloroglucinol: antioxidant properties and effects on cellular oxidative markers in human HepG2 cell line. Food Chem. Toxicol..

[82] Kang KA, Lee KH, Chae S, Zhang R, Jung MS, Ham YM (2006). Cytoprotective effect of phloroglucinol on oxidative stress induced cell damage via catalase activation. J. Cell. Biochem..

[83] So MJ, Cho EJ (2014). Phloroglucinol attenuates free radical-induced oxidative stress. Prev. Nutr. Food Sci..

[84] Dell'Orco M, Milani P, Arrigoni L, Pansarasa O, Sardone V, Maffioli E (2016). Hydrogen peroxide-mediated induction of SOD1 gene transcription is independent from Nrf2 in a cellular model of neurodegeneration. Biochim. Biophys. Acta.

[85] Chiang SK, Chen SE, Chang LC (2021). The role of HO-1 and its crosstalk with oxidative stress in cancer cell survival. Cells.

[86] Leiser SF, Miller RA (2010). Nrf2 signaling, a mechanism for cellular stress resistance in long-lived mice. Mol. Cell. Biol..

[87] Suzuki M, Otsuki A, Keleku-Lukwete N, Yamamoto M (2016). Overview of redox regulation by Keap1-Nrf2 system in toxicology and cancer. Curr. Opinion Toxicol..

[88] Ohn J, Kim SJ, Choi SJ, Choe YS, Kwon O, Kim KH (2018). Hydrogen peroxide (H_2_O_2_) suppresses hair growth through downregulation of β-catenin. J. Dermatol. Sci..

[89] Huang WY, Huang YC, Huang KS, Chan CC, Chiu HY, Tsai RY (2017). Stress-induced premature senescence of dermal papilla cells compromises hair follicle epithelial-mesenchymal interaction. J. Dermatol. Sci..

[90] Vañó-Galván S, Camacho F (2017). New treatments for hair loss. Actas Dermosifiliogr..

[91] Taghiabadi E, Nilforoushzadeh MA, Aghdami N (2020). Maintaining hair inductivity in human dermal papilla cells: a review of effective methods. Skin Pharmacol. Physiol..

[92] Woo WM, Zhen HH, Oro AE (2012). Shh maintains dermal papilla identity and hair morphogenesis via a Noggin-Shh regulatory loop. Genes Dev..

[93] Hwang SB, Park HJ, Lee BH (2022). Hair-growth-promoting effects of the fish collagen peptide in human dermal papilla cells and C57BL/6 mice modulating Wnt/β-catenin and BMP signaling pathways. Int. J. Mol. Sci..

[94] Deng Z, Chen M, Liu F, Wang Y, Xu S, Sha K (2022). Androgen receptor-mediated paracrine signaling induces regression of blood vessels in the dermal papilla in androgenetic alopecia. J. Invest. Dermatol..

[95] Kwack MH, Sung YK, Chung EJ, Im SU, Ahn JS, Kim MK (2008). Dihydrotestosterone-inducible dickkopf 1 from balding dermal papilla cells causes apoptosis in follicular keratinocytes. J. Investig. Dermatol..

[96] Kwack MH, Ahn JS, Kim MK, Kim JC, Sung YK (2012). Dihydrotestosterone-inducible IL-6 inhibits elongation of human hair shafts by suppressing matrix cell proliferation and promotes regression of hair follicles in mice. J. Invest. Dermatol..

[97] Ruksiriwanich W, Khantham C, Muangsanguan A, Phimolsiripol Y, Barba FJ, Sringarm K (2022). Guava (*Psidium guajava* L.) leaf extract as bioactive substances for anti-androgen and antioxidant activities. Plants (Basel, Switzerland).

[98] Shin JY, Choi YH, Kim J, Park SY, Nam YJ, Lee SY (2020). Polygonum multiflorum extract support hair growth by elongating anagen phase and abrogating the effect of androgen in cultured human dermal papilla cells. BMC Complement. Med. Ther..

[99] Choi YH, Shin JY, Kim J, Kang NG, Lee S (2021). Niacinamide down-regulates the expression of DKK-1 and protects cells from oxidative stress in cultured human dermal papilla cells. Clin. Cosmet. Investig. Dermatol..

[100] Yang F, Cao Y (2012). Biosynthesis of phloroglucinol compounds in microorganisms--review. Appl. Microbiol. Biotechnol..

[101] Zhou L, Xu M, Yang Y, Yang K, Wickett RR, Andl T (2016). Activation of β-catenin signaling in CD133-positive dermal papilla cells drives postnatal hair growth. PLoS One.

[102] Zhao S, Fu J, Liu X, Wang T, Zhang J, Zhao Y (2012). Activation of Akt/GSK-3beta/beta-catenin signaling pathway is involved in survival of neurons after traumatic brain injury in rats. Neurol. Res..

[103] Tang Z, Yang G, Wang X, Chen F, Liao Z, Zhang Z (2020). AKT/GSK-3β/β-catenin signaling pathway participates in erythropoietin-promoted glioma proliferation. J. Neurooncol..

[104] Fukumoto S, Hsieh CM, Maemura K, Layne MD, Yet SF, Lee KH (2001). Akt participation in the Wnt signaling pathway through Dishevelled. J. Biol. Chem..

[105] Prie BE, Voiculescu VM, Ionescu-Bozdog OB, Petrutescu B, Iosif L, Gaman LE (2015). Oxidative stress and alopecia areata. J. Med. Life.

[106] Prie BE, Iosif L, Tivig I, Stoian I, Giurcaneanu C (2016). Oxidative stress in androgenetic alopecia. J. Med. Life.

[107] Upton JH, Hannen RF, Bahta AW, Farjo N, Farjo B, Philpott MP (2015). Oxidative stress-associated senescence in dermal papilla cells of men with androgenetic alopecia. J. Invest. Dermatol..

[108] Gaff AN A-, S H, SAW (2005). Effect of melatonin on oxidative stress markers in patients with alopecia areata. Iraq. J. Pharm..

[109] Shakoei S, Mirmiranpoor H, Nakhjavani M, Nasimi M, Bakhshi G, Azizpour A (2023). Oxidative stress and antioxidant markers in patients with alopecia areata: A comparative cross-sectional study. Indian J. Dermatol. Venereol. Leprol..

[110] Acharya P, Mathur MC (2020). Oxidative stress in alopecia areata: a systematic review and meta-analysis. Int. J. Dermatol..

[111] Haslam IS, Jadkauskaite L, Szabó IL, Staege S, Hesebeck-Brinckmann J, Jenkins G (2017). Oxidative damage control in a human (Mini-) Organ: Nrf2 activation protects against oxidative stress-induced hair growth inhibition. J. Invest. Dermatol..

[112] Park C, Cha HJ, Kim MY, Bang E, Moon SK, Yun SJ (2022). Phloroglucinol attenuates DNA damage and apoptosis induced by oxidative stress in human retinal pigment epithelium ARPE-19 cells by blocking the production of mitochondrial ROS. Antioxidants (Basel, Switzerland).

