# Supplementary material for: Phloroglucinol Enhances Anagen Signaling and Alleviates H2O2-Induced Oxidative Stress in Human Dermal Papilla Cells
Source: J Microbiol Biotechnol. 2024 Mar 8;34(4):812–27. doi: 10.4014/jmb.2311.11047 (PMC11091678; doi:10.4014/jmb.2311.11047)
Supplement: Supplementary file 1 [file jmb-34-4-812-supple.pdf]

J. Microbiol. Biotechnol.
https://doi.org/10.4014/jmb.2311.11047

Supplementary Figures

Phloroglucinol Enhances Anagen Signaling and Alleviates H_2O_2-Induced Oxidative Stress in Human Dermal Papilla Cells

Seokmuk Park[1], Ye Jin Lim[1], Hee Su Kim[1], Hee-Jae Shin[1], Ji-Seon Kim[1], Jae Nam Lee[2], Jae Ho Lee[1] and Seunghee Bae[1*]

[1]*Department of Cosmetics Engineering, Konkuk University, 120 Neungdong-ro, Gwangjin-gu, Seoul 05029, Republic of Korea*

[2]*Department of Cosmetology, Graduate School of Engineering, Konkuk University, 120 Neungdong-ro, Gwangjin-gu, Seoul 05029, Republic of Korea*

*Corresponding author: Seunghee Bae

E-mail: sbae@konkuk.ac.kr

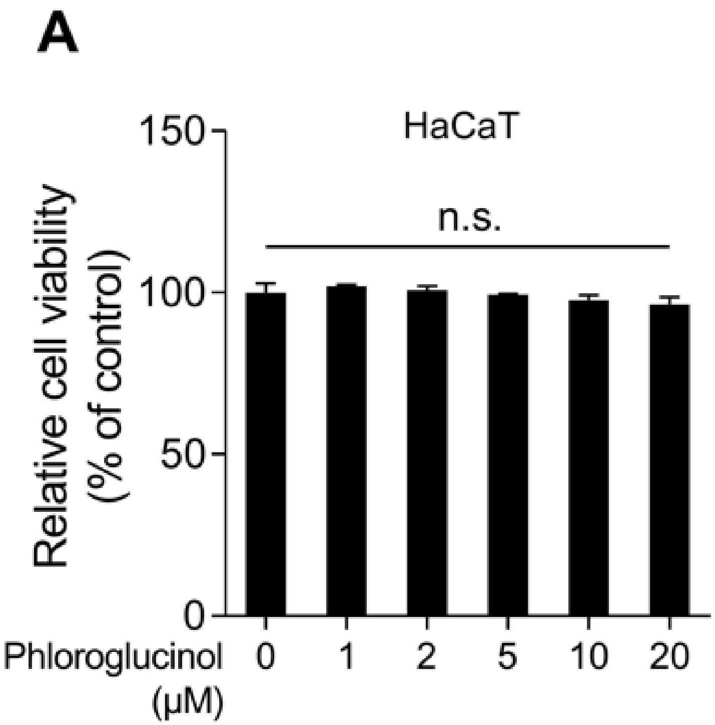

Fig. S1. **Effects of phloroglucinol (PG) on cell viability in HaCaT cells**. **(A)** HaCaT cells are treated with PG (0-20 μM) for 48 h, and cell viabilities are assessed using a WST-1 assay. The results are presented as the mean ± SD of three independent experiments and are analyzed using a one-way analysis of variance followed by Tukey's test.

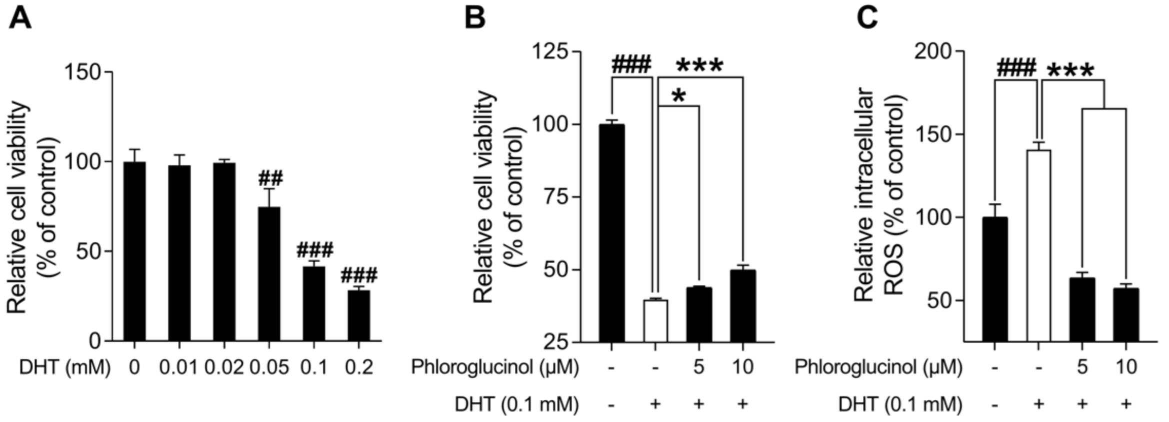

Fig. S2. **Effects of phloroglucinol (PG) on dihydrotestosterone (DHT)-induced cytotoxicity in HDPCs. (A)** HDPCs are treated with the indicated concentrations of DHT (0-0.2 mM) for 48 h, and cell viabilities are assessed using a WST-1 assay. (B) The decreased toxicity of HDPCs treated with PG against DHT-stimulation. HDPCs are exposed to indicated concentrations of PG (0-10 μM) for 30 min followed by treatment with 0.1 mM DHT for 48 h. The cell viabilities are assessed using a WST-1 assay. (C) Intracellular ROS levels in HDPCs are assessed using a DCF-DA microplate reader assay. The results are presented as the mean ± SD of three independent experiments and are analyzed using a one-way analysis of variance followed by Tukey's test. $^{##}p < 0.01$; $^{###}p < 0.001$ compared with the vehicle-treated group. $^{*}p < 0.05$; $^{***}p < 0.001$ compared with the 0.1 mM H_2O_2-treated group.
